# National Public Health Surveillance of Corporations in Key Unhealthy Commodity Industries – A Scoping Review and Framework Synthesis

**DOI:** 10.34172/ijhpm.2023.6876

**Published:** 2023-03-05

**Authors:** Elizabeth Bennett, Stephanie M. Topp, Alan Rob Moodie

**Affiliations:** ^1^College of Public Health, Medical and Veterinary Sciences, James Cook University, Townsville, VIC, Australia; ^2^Melbourne School of Population and Global Health, University of Melbourne, Melbourne, VIC, Australia

**Keywords:** Commercial Determinants, Public Health Surveillance, Monitoring, Unhealthy Commidity Industries, Corporate Influence

## Abstract

**Background:** Corporations in unhealthy commodity industries (UCIs) have growing influence on the health of national populations through practices that lead to increased consumption of unhealthy products. The use of government-led public health surveillance is best practice to better understand any emerging public health threat. However, there is minimal systematic evidence, generated and monitored by national governments, regarding the scope of UCI corporate practices and their impacts. This study aims to synthesise current frameworks that exist to identify and monitor UCI influence on health to highlight the range of practices deployed by corporations and inform future surveillance efforts in key UCIs.

**Methods:** Seven biomedical, business and scientific databases were searched to identify literature focused on corporate practices that impact human health and frameworks for monitoring or assessment of the way UCIs impact health. Content analysis occurred in three phases, involving (1) the identification of framework documents in the literature and extraction of all corporate practices from the frameworks; (2) initial inductive grouping and synthesis followed by deductive synthesis using Lima and Galea’s ‘vehicles of power’ as a heuristic; and (3) scoping for potential indicators linked to each corporate practice and development of an integrated framework.

**Results:** Fourteen frameworks were identified with 37 individual corporate practices which were coded into five different themes according the Lima and Galea ‘Corporate Practices and Health’ framework. We proposed a summary framework to inform the public health surveillance of UCIs which outlines key actors, corporate practices and outcomes that should be considered. The proposed framework draws from the health policy triangle framework and synthesises key features of existing frameworks.

**Conclusion:** Systematic monitoring of the practices of UCIs is likely to enable governments to mitigate the negative health impacts of corporate practices. The proposed synthesised framework highlights the range of practices deployed by corporations for public health surveillance at a national government level. We argue there is significant precedent and great need for monitoring of these practices and the operationalisation of a UCI monitoring system should be the object of future research.

## Introduction

 Unhealthy commodity industries (UCIs) have growing influence on the health of national populations through corporate practices that lead to increased consumption of unhealthy products.^1-6^ Harmful consumption of ultra-processed foods and beverages, tobacco and alcohol are central to the rise of non-communicable diseases (NCDs) which now account for more than two-thirds of the global burden of disease.^3,7-10^ Corporations within UCIs are defined as corporations where a significant share of their product portfolio comprises unhealthy commodities with high profit margins aimed at, and easily accessible to, large numbers of consumers.^3,11,12^ Corporate activities that promote and defend these behaviours can be found across different types of unhealthy industries and pose a risk to the development and implementation of effective policies for NCD control.^13,14^ While there is increasing recognition of the ways that corporations influence health, there is minimal systematic surveillance of the scope of these practices and their impacts at a national government level. An increasing understanding of UCI activities as a composite of risk factors for NCDs can enable integrated strategies for NCD prevention.^15^ The use of government-led public health surveillance is best practice to better understand any emerging public health threat, however there is no accepted comprehensive framework for national governments to monitor unhealthy commodity corporate practices.^9,16-18^ This information is crucial to inform effective programs to curtail the role of these industries as key inducers of NCDs.^19,20^

## Rationale

 Public health surveillance is the continuous, systematic collection and interpretation of health-related data needed for the planning, implementation and evaluation of public health practice.^21^ Evidence indicates that a strong regulatory framework, including monitoring, is needed to mitigate the negative health impacts of corporations.^2,22-27^ There is also evidence that monitoring and accountability systems could better facilitate public health and private sector engagement on NCDs by ensuring safeguards are in place to define engagement and manage potential conflicts of interest.^25^ Despite this, systematic monitoring of unhealthy corporate influence on health is largely absent at a national government level, and there is no established framework of surveillance that can be used across industries to monitor and evaluate these impacts. The lack of a comprehensive framework may reflect the inherent complexity in implementing feasible and acceptable interventions, particularly given the need for inter- disciplinary coordination. Governing NCDs also frequently brings public health into conflict with the interests of the powerful and highly influential profit-driven food, beverage, alcohol and tobacco industries.^11,26^

 The number, reach and power of corporations has grown exponentially over the past few decades.^2,28^ Many transnational corporations now have economies that are larger than those of nation states.^29^ Indeed, our analysis of World Bank and Capital IQ data to identify the top 100 governments and corporations with the highest annual revenues in 2019 demonstrate 75 were corporations, an increase from 71 in 2016.^30-32^ Growth of these enterprises is facilitated by the broader global context, in which neoliberal capitalist policies promoting trade liberalisation and producer subsidies, and increasing demand for products in low- and middle-income countries (LMICs) are all features.^3,29,33^ It is important to acknowledge that corporations are not a homogenous entity and have the capacity to positively contribute to society, and health.^29,34^ However, by the nature of the products they produce and the profit motive that underpins their operation, unhealthy commodities corporations have a detrimental impact on health.^17^ There is also increasing evidence that tactics employed by some UCIs are being taken up more broadly.^10^ For example, strategies long employed by the tobacco industry, such as denial of science, are now seen in the food and beverage and alcohol industries.^2,5^ There is an increasing call for intersectoral policy on UCIs which provides an opportunity for the development public health responses that can be used across industries, including monitoring systems.^35,36^ We recognise there is growing acknowledgement in the literature that the public health harms of commercial actors span to other industries, including pharmaceuticals, firearms, social media and financial institutions, and go beyond commodities to practices and use of power. We have decided to focus on select industries for this scoping review for two reasons, (1) the selected industries have been the focus of the public health scientific community to date and therefore have a more robust documentation of practices,^2,37^ and (2) to limit the scope of this initial review in the context of time and resourcing parameters.

## Objectives

 This study aims to synthesise current frameworks that exist to identify and monitor UCI influence on health to create a template surveillance system to be used by national governments across industries. Strengthening the capacity of governments to investigate and monitor these industries can equip public health decision makers to develop effective policy.^2^ This research builds on the seminal work of a number of public health academics and advocates who have developed frameworks identifying and assessing UCI practices that impact health. However, to date these have tended to focus on particular industry or type of corporate activity, for example Swinburn et al developed an approach to monitor UCI practices related to food environments.^2,6,8,37^ This now forms part of the International Network for Food and Obesity/NCD Research, Monitoring and Action Support monitoring of food industry policies, and has been instrumental in assessing the commitments of food and beverage corporations.^37^ Yet so far this monitoring system has not been applied to the UCI activities of other industries such as tobacco.^39^ McCambridge et al also performed a systematic review of public health surveillance studies of alcohol industry actors that explore the implications of business and political strategies for health.^18^ As above, however, industry-specific nature of the review may have resulted in identification of industry-specific practices. Mialon et al, too have proposed a framework for categorizing and monitoring the corporate political activity of the food industry, which has been implemented in over 20 countries.^40^ But again, since the framework focuses on the food industry and the corporate political activity of corporations, there is the potential for UCI practices outside of this scope to be missed.

 While there are some frameworks that identify a range of corporate activities across industries, the majority of these have been designed or proposed for use by researchers or civil society, instead of by governments as a part of routine public health surveillance. Wood et al, for example, proposed a ‘Corporate Power and Health’ framework to inform analysis of commercial determinants of health, however this framework focuses on the conceptual integration of power theories into key features of existing commercial determinants of health frameworks, rather than their use for public health surveillance.^41^ Additionally, Baum et al proposed an approach to assessing the health impact of transnational corporations.^29^ This “Corporate-Health Impact Assessment” has been implemented for a number of UCIs, however this was thought to be most likely used by civil society activists with academic research support.^23,29^ There is also not currently, to the authors’ knowledge, a global consensus regarding what practices should be prioritised for monitoring.^9,40,42^ As such, there is a paucity of evidence regarding the barriers and enablers to implementation of multi-industry surveillance of UCIs at a national government level. Overall, this research seeks to lay a foundation for the development and implementation of a preliminary multi-industry monitoring framework that can be adapted by national governments to monitor and mitigate the impacts of corporate activities and reduce the burden of NCDs.

## Methods

 A scoping review of the academic literature was conducted to identify previous efforts to identify and monitor corporate impact on health through the production, promotion and consumption of harmful commodities such as ultra-processed foods and beverages, tobacco and alcohol. The review was completed according to Preferred Reporting Items for Systematic Reviews and Meta-Analyses (PRISMA) guidelines and identified documents were qualitatively examined using content analysis and a framework synthesis approach was used to create a framework to inform public health surveillance of corporations in key UCIs.^43^

###  Eligibility Criteria 

 To be included in this review, publications had to:

- Be published in 2000 or later.
- Be published a peer-reviewed journal in the English language.
- Be conceptualised as studies examining the way corporate practices impact human health.
- Propose a new framework for monitoring or assessment of the way UCIs impact health.
- Focus on the influence of the tobacco, alcohol and ultra-processed foods on health.

###  Information Sources and Search Strategy 

 A scoping search was conducted on Web of Science (Web of Science Interface), MEDLINE (Ovid interface), Embase (Ovid interface), PsycINFO (Ovid interface), Scopus (Scopus interface), Business Source Premier (EBSCOhost interface) and CINAHL (EBSCOhost interface). Each database was searched from 2000 onward and were last searched on October 2, 2021. The initial search strategy used included review of public health, social science and business databases, with the search terms [corporat* OR commercial], [health OR public health] [influence OR impact OR tactic OR strategy] [surveillance OR monitor*]. The search strategy was organised around the three constructs of “corporation,” “public health” and “surveillance” and was developed with the support of a specialist librarian. This resulted in the identification of 2464 articles, 855 of which were duplicates. This was supplemented by snowball searching to identify additional documents in citation searches and grey literature.

###  Selection and Data Collection Process 

 The material retrieved was downloaded, imported into EndNote and duplicates were removed using this software. Titles and abstracts were screened by EB, and potentially eligible full texts were obtained. Data extracted included authors, year, framework title, industry, method of developing framework and main findings. We sorted the identified papers in order of the year they were published and coded them according to whether the framework document identified corporate practices as described in the developed framework below.

###  Content Analysis and Synthesis 

 Content analysis was completed in three phases; in the first phase of inductive coding, text describing corporate practices was extracted into an undifferentiated list of practices. No particular framework or theory was used to guide the extraction.

 In the second phase we looked at the list of practices to identify commonalities in type and purpose of corporate practices. Two authors individually developed ideas for grouping practices and came together to discuss these. Examples of initial ideas for groupings included, for example, *marketing strategies; political influence; legal and regulatory strategies. *As part of the discussions, the Madureira Lima and Galea ‘vehicles of power’ framework was discussed.^17^ A consensus was reached that the five vehicles of power was an appropriate heuristic for organising the extracted corporate practices due to the recognised usefulness of those domains for thinking strategically (rather than just descriptively) about how corporate practices can be measured and monitored. As part of this decision, particular attention and consideration were given to practices not included in Lima and Galea’s own framework to establish whether new or additional domains were warranted.^17^ Phase three involved exploring the relevance of the identified corporate practices to policy-makers; scoping potential indicators, and developing an integrated framework. As part of exploring the relevance of these corporate practices to policy-makers, we adapted elements of the Walt and Gilson Health Policy Triangle to highlight key actors that should be considered in the process of surveillance, and practices and outcomes that should be monitored to inform policy to prevent negative impacts of UCIs.^44^ We proposed an synthesised framework for surveillance of corporations in key UCIs by integrating the framework for analysis with the synthesis of identified framework documents.

## Results

###  Review Findings

**Figure 1:**
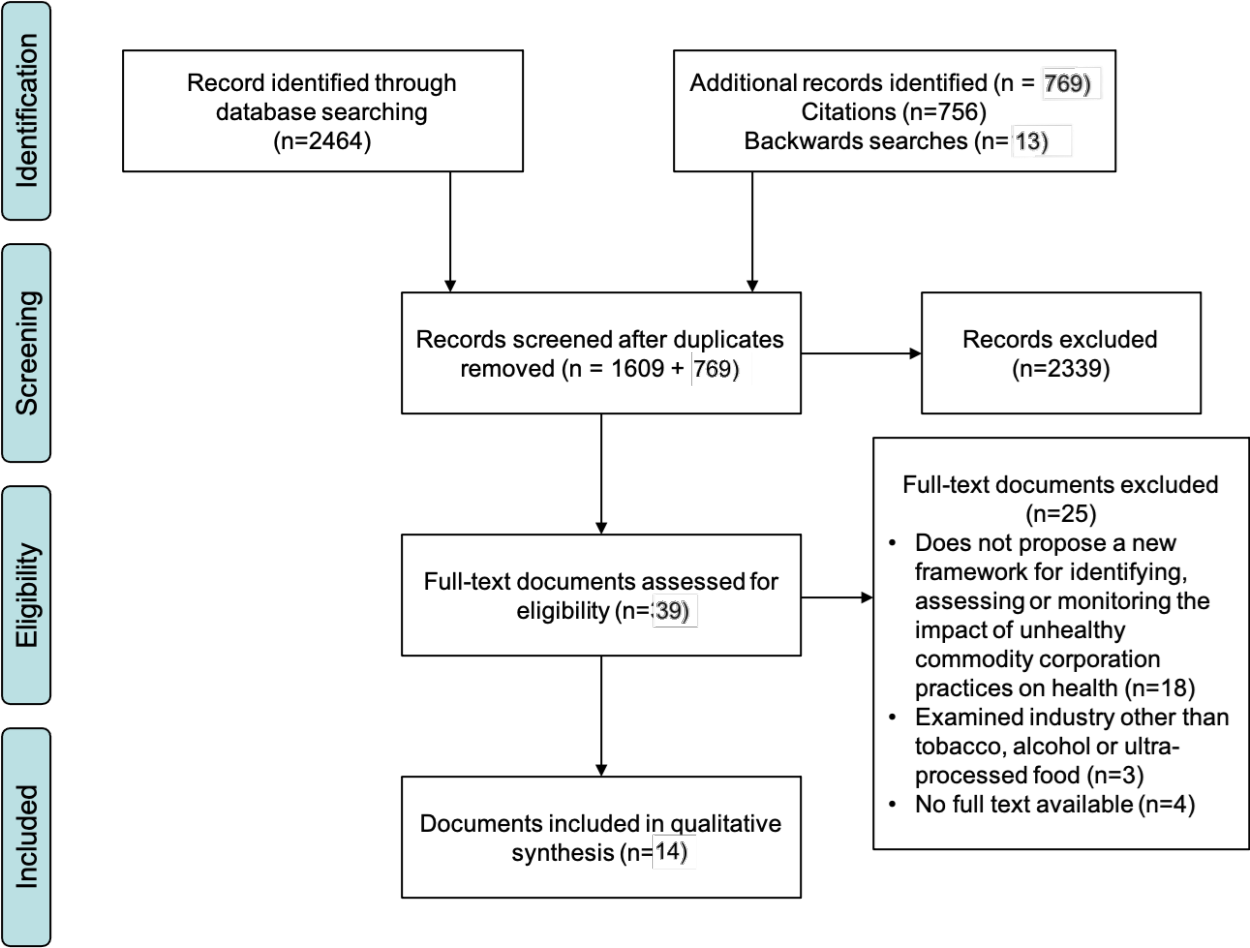
PRISMA Flow Diagram. Abbreviation: PRISMA, Preferred Reporting Items for Systematic Reviews and Meta-Analyses.

 We identified 14 frameworks or framework reviews designed to identify or monitor how corporate practices influenced health outcomes that met eligibility criteria (refer to Figure 1 for corresponding PRIMSA diagram). These studied corporate activity that impacts health across the UCIs of tobacco,^45-47^ alcohol^18^ and ultra-processed food^35,37^ and the monitoring of the impact of corporate practices on health across industries.^9,17,36,41,42,48,49^ Two studies, Trochim et al and Stillman et al, were regionally specific to North America and Asia, respectively. Where multiple studies existed that included the identified framework, the original article was included in the review.^45,46^

 The studies used a range of methods to develop frameworks for assessing and monitoring corporate impact on health, including concept mapping, brainstorming, theory-based adaptation and systematic review (see Table 1). Thirty-seven corporate practices that impact health were identified for monitoring with some included in more frameworks than others (see Table 2).

**Table 1:** Identified Frameworks for Assessing and Monitoring Corporate Impact on Health

**First Author (Year)**	**Framework**	**Industry**	**Method**	**Findings**
Trochim^46^ (2003)	American Stop Smoking Intervention Study Model	Tobacco	Concept mapping	Identified four overarching practice clusters: science public relations, issue framing, harassment, and lobbying tactics.
Wiist^42^ (2006)	Recommendations for linking public health and the “anti-corporate movement”	All	Expert opinion	Identified 18 measures relating to activity of the corporate entity and 16 public health status indicators.
Jahiel^9^ (2008)	Epidemiologic cascade framework	All	Theory based- adaptation of the agent-host-environment model	Five levels of analysis identified: government, corporations, corporate conduits, the environment of the host, and the host itself. Multiple objects of surveillance identified at each level.
Stillman^45^ (2008)	Mapping tobacco industry strategies in Southeast Asia	Tobacco	Concept mapping	Eight clusters of industry activities within 4 Southeast Asian countries arranged into four sectors: economics (avoid economic regulation, business/investment strategies), politics (lobbying/political influence, silence/reduce opposition), public relations (self-serving industry youth programmes, marketing tactics/image building) and deception (deceiving public, corrupting and manipulating science).
Sacks^37^ (2013)	Business impact assessment - obesity	Food	Theory based- adapted from Access to Nutrition Index	Evaluation of corporations’ commitment to reducing obesity/NCD in the following domains: corporate strategy, relationships with external organisations, product formulation, nutrition labelling, promotion to children/adolescents and product accessibility.
Baum^29^ (2015)	Corporate health impact assessment	All	Brainstorming	Identifies three tiers of transnational corporations impact within a country: context (corporate structure, status, political environment, regulatory capacity), structure (size, operational structure including political and business practices, products, distribution, marketing) and health impact (occupational health, social conditions, natural environment, health related behaviours, economic conditions).
Mialon^40^ (2015)	Corporate political activity assessment	Food	Theory based-adaptation of Savell taxonomy of corporate political activity	Six overarching strategies of corporate political activity identified: information and messaging, financial incentive, constituency building, legal, policy substitution, opposition fragmentation and destabilisation. Within each strategy multiple practices and mechanisms were identified.
Ulucanlar^47^ (2016)	Policy Dystopia Model	Tobacco	Thematic analysis of literature using grounded theory approach	Developed taxonomy of instrumental strategies and techniques used by the tobacco industry.
Knai^48^ (2018)	Systems thinking framework	All	Theory based- adaption of systems thinking proposed by Meadows	Identifies market and nonmarket components of UCI strategies. Examines the NCD-genic systems using elements (actors, market access/trade, consumption patterns), interconnections (physical/information flows) and purpose (goals of corporation and government).
Madureira Lima^17^ (2018)	Three-dimensional view of power	All	Theory based- Adaptation of Steven Luke’s three-dimensional view of power	Identifies five vehicles of power used by UCIs: political environment, preference shaping, knowledge environment, legal environment and extra-legal environment.
McCambridge^18^ (2019)	Systematic review of public health surveillance studies of the alcohol industry	Alcohol	Systematic review	Identified 6 frameworks for public health surveillance of alcohol industry market and political strategies.
Keshavarz Mohammadi^49^ (2020)	OSRAH	All	Literature review and focus group discussion	Developed conceptual framework organisation social responsibility and accountability for health which outlined five domains of OSRAH and six aspects of organisations. A tool for assessment against the conceptual framework was also developed and used to assess 95 organisations at a national conference in Iran.
Wood^41^ (2021)	Narrative review of commercial determinants of health frameworks	All	Narrative review and synthesis	Identified 22 frameworks describing commercial determinants of health and synthesised to incorporate theories of power.
Legg^36^ (2021)	The Science for Profit Model	All	Scoping review and interpretive analysis	Identified eight corporate sectors repeatedly engaging in activities to influence science, including manipulation of scientific methods; reshaping of criteria for establishing scientific “proof”; threats against scientists’ and clandestine promotion of policy reforms that increase reliance on industry evidence.

Abbreviations: UCI, unhealthy commodity industry; NCD, non-communicable disease; OSRAH, Organisational Social Responsibility and Accountability for Health.

**Table 2:** Practices of Unhealthy Commodity Industries With Influence on Health Identified in Existing Monitoring Frameworks

	**Trochim** ^46^	**Wiist** ^42^	**Jahiel** ^9^	**Stillman** ^45^	**Sacks** ^37^	**Baum** ^50^	**Mialon** ^40^	**Ulucanlar** ^47^	**Knai** ^51^	**Madureira Lima** ^17^	**McCambridge** ^18^	**Keshavarz Mohammadi** ^49^	**Wood** ^41^	**Legg** ^36^
**Political environment**														
Lobbying	**•**	**•**	**•**	**•**	**•**	**•**	**•**	**•**		**•**	**•**		**•**	**•**
Political donations	**•**	**•**	**•**	**•**	**•**		**•**	**•**	**•**	**•**	**•**		**•**	
Direct participation in policy-making via government agencies and partnerships	**•**	**•**	**•**	**•**			**•**	**•**	**•**	**•**	**•**		**•**	**•**
Revolving doors			**•**				**•**			**•**			**•**	
Policy substitution	**•**			**•**			**•**						**•**	
Promote self-regulation or de-regulation	**•**		**•**	**•**	**•**			**•**	**•**				**•**	
Involvement in international trade negotiations		**•**		**•**		**•**	**•**			**•**	**•**		**•**	**•**
Pressures on national governments				**•**		**•**	**•**	**•**					**•**	
Tax avoidance				**•**						**•**				
Tied development aid										**•**				
Pressures on international organisations				**•**						**•**				
Portfolio diversification				**•**										
**Preference Shaping**														
Corporate social responsibility	**•**	**•**	**•**	**•**	**•**	**•**	**•**		**•**	**•**	**•**	**•**	**•**	
Marketing and advertising	**•**	**•**	**•**	**•**	**•**	**•**			**•**	**•**	**•**		**•**	
Product modification and targeting vulnerable populations	**•**		**•**	**•**	**•**	**•**							**•**	
Product amount and concentration			**•**	**•**	**•**	**•**			**•**		**•**			
Pricing			**•**	**•**	**•**					**•**	**•**			
Product Availability			**•**		**•**	**•**			**•**		**•**		**•**	
Civil society capture	**•**			**•**	**•**					**•**			**•**	**•**
Capturing of the media	**•**						**•**			**•**			**•**	**•**
Use of public relations companies			**•**					**•**		**•**				
Key opinion leaders and funding health organisations	**•**			**•**			**•**	**•**		**•**				**•**
Manufacturing doubt	**•**		**•**	**•**			**•**	**•**	**•**	**•**	**•**		**•**	**•**
Issue framing and attention deflection	**•**		**•**				**•**		**•**	**•**				**•**
Building business coalitions	**•**		**•**	**•**	**•**	**•**	**•**		**•**					**•**
**Knowledge environment**														
Funding research/academic institutions	**•**		**•**	**•**	**•**		**•**	**•**	**•**	**•**			**•**	**•**
Industry sponsored education	**•**			**•**			**•**			**•**	**•**		**•**	**•**
Scientific advisory boards/science institutes	**•**						**•**	**•**		**•**				**•**
Suppress publication of unfavourable science														**•**
**Legal environment**														
Litigation and pre-emption	**•**		**•**			**•**	**•**	**•**		**•**	**•**		**•**	**•**
Liability	**•**									**•**			**•**	**•**
Unregulated activity/externalised costs		**•**								**•**				
Using international activities to avoid domestic regulation	**•**			**•**		**•**								
**Extra-legal environment**														
Corporate illegal activity	**•**			**•**	•			•	•	**•**	•		**•**	
Harassment	**•**			**•**		**•**		•					**•**	**•**
Opposition fragmentation	**•**			**•**			•	•		**•**				
Tax evasion						**•**				**•**				

 The Madureira Lima and Galea framework was found to incorporate 26 out of 37 corporate practices categorised according to five major domains: political environment, preference shaping, knowledge environment, legal environment and extra-legal environment.^17^

###  Political Environment

 Practices included within the political environment included lobbying, political donations, direct participation in government agencies, partnerships and policy development, revolving doors, involvement in international trade negotiations, policy substitution, promotion of self-regulation and de-regulation, tied development aid, pressure on international organisations, tax avoidance and portfolio diversification. Corporations in UCIs exert undue influence through lobbying by gaining access to policy-makers in a way unavailable to other individuals and organisations who do not have the same resources to invest.^17^ Furthermore, the information that corporate lobbyists share with government often carries weight as expert information, even though it may be biased, incomplete or erroneous.^4,17^ Political donations enable favourable decision-making and political agenda-setting because reciprocity may be expected once the party is in office.^51,52^ Corporations whose interests may conflict with the public’s may protect and expand their activities by becoming partners in the formulation of public policy, often leading to weakening of public health policies.^17,53^ Revolving doors refer to the flow of employees between the public and private sector (at national and international levels) and can advance corporate interests by favouring industry interests in policy decisions, access to confidential information and guaranteeing industry voice in the policy-making process.^54^ Corporations in UCIs can also suggest alternate policy to be substituted in the place of evidence-based public health initiatives, for example, by promoting partial or weak measures as an alternative to effective measures or inserting limiting language in legislation.^41,45,46,55^ Corporations may also promote self-regulation or deregulation to avoid legislative interventions. Self-regulation can seek to usurp the public health process by adopting voluntary codes and establishing non-regulatory initatives.^41,45,46,48^ Promotion of de-regulation can be used to shape the narrative and public perceptions about the role that governments should play (eg, nanny-statism). Corporate pressures on trade agreements also can influence health through increased availability and decreased prices of unhealthy commodities.^56,57^ Corporations in UCIs have also been noted to use their structural power relative to national governments to apply pressure or threaten shifting of jobs, production processes, capital and support if undesirable regulations were to be implemented.^29,41,45,47,55^ Tax avoidance leads to a shortage of public tax revenue to be directed for health and social purposes.^45,58^ Corporations in UCIs shape health of populations in the developing world by tying aid to purchases from corporations in donor countries, acting as de facto export promotion.^17^ They also exert pressure on international organisations by providing financial support to key institutions as International Monetary Fund and the World Health Organization (WHO).^17,45^ They may also protect their interests via governmental representatives or directly via participants in delegations to international bodies with mandates to regulate their activities.^17,59^ Portfolio diversification (for example, an ultra-processed food corporation entering into alcohol and clothing businesses) can be used by corporations to protect themselves economically.^45^ This practice is considered to act within the political environment as it may be used to increase the economic power of corporations and increase their ability to leverage this power in policy or regulatory context.^45^

 Within this group of practices direct participation in government agencies, partnerships and policy development, lobbying and donations were identified as monitoring priorities in eleven or more of the fourteen frameworks.

 A range of potential indicators for monitoring UCI corporate activity in the political environment were identified and are listed in full in Table S1. Examples include the number of direct and indirect registered lobbyists^40^; the number of UCI representatives on national policy committees^9^; evaluation of the number of trade agreements which favour corporations.^60^ Sources of data for these indicators were more disparate and likely to vary considerably country to country. Some research identified the role of direct requests under freedom of information legislation while others suggested harvesting data from public websites and documents.^55,61,62^ Organisations that are doing existing work in this area include Corporate Observatory Europe who produce narrative reports on topics such as a “Revolving Door Watch” and highlighting lobbying in the European Commission.^63^ This monitoring appears to be effective as evidence used in campaigning for greater lobbying transparency, however it is unclear if this would be useful for government level surveillance purposes.^64^ Furthermore, the Framework Convention of Tobacco Control (FCTC) Article 5.3 provides guidelines for monitoring of government interaction with the tobacco industry, including industry participation in partnerships and policy development and political contributions by tobacco corporations.^65^ However, as this is self-reported by national governments there are significant discrepancies in reporting standards.^66^ This was also identified as an ongoing challenge to implementation of FCTC in a global evidence review prepared for the Impact Assessment Expert Group.^67^ Several non-governmental organisations produce country reports according to the FCTC guidelines, including the Global Tobacco Industry Interference Index. This initiative provides country level reports for 57 countries which examine compliance with FCTC Article 5.3 guidelines, and assesses countries’ response to tobacco industry interference and protecting their public health policies from interference^68^ (see Table S1 in Supplementary file 1).^17,29,36,37,40-42,45-48,50,52,53,55-58,69-90^

###  Preference Shaping

 The preferencing shaping strategies identified included corporate social responsibility, marketing and advertising, product modification and targeting vulnerable populations, issue framing and attention deflection, product amount and concentration, pricing and product availability, civil society capture, capturing of the media, use of public relations companies, key opinion leaders and funding health organisations, manufacturing doubt, manufacturing disease, leveraging business afflations.^17^ *Corporate social responsibility *refers to a concept of business self-regulation with the aim of integrating social or environmental concerns in business operations.^91^ It can influence health by increasing exposure to unhealthy products that would otherwise be more tightly regulated and expanding corporate marketing reach and product acceptability through association with the image of social commitment.^75,76^ This can include practices such as corporate philanthropy, public private partnerships, and corporate aid programs, such as tobacco companies funding shelters for victims of domestic violence.^77^ Higher levels of public health research into the impact of unhealthy industries and higher per capita consumption of unhealthy products have been associated with increased prevalence of these practices suggesting that they may be implemented as a way to preserve markets by counteracting scientific evidence of related harms.^78^ Corporate strategies around *product modification* and *targeting of vulnerable populations* such as youth, women or low-income groups aim to increase consumption. For example, Esser and Jernigan found that in regions experiencing economic development, global alcohol corporations tend to seek opportunities to expand their consumer base through increased use of marketing strategies that appeal to groups that typically have lower rates of drinking, such as youth and women.^79^ *Marketing and advertising* expand the number of consumers of unhealthy products and shapes the social acceptability of commodities.^17,81^ Monitoring of marketing practices, particularly those in breach of codes or legislation to minimise, has been identified as an important strategy to reduce exposure of unhealthy products to particular groups.^92^ *Product amount and concentration*, including the design of products to maximise consumption eg, through increased amount of salt to improve taste – influences health through increasing the amount of unhealthy substances are available for consumption.^9,86^ Another relevant strategy within product amount and concentration is *reformulation*. There is increasing evidence that corporations position product reformulation as a benevolent public health response, despite being strongly underpinned by business and political incentives, such as improving corporate public image and minimising the threat of mandatory regulation.^93^ *Pricing*, too, enables the sale of greater quantities of unhealthy commodities and increases availability to low-income groups.^17,82^ *Product availability* (where the product is available including the number of retail units (eg, vending machines, restaurants, bars, supermarkets), location and timing of sales) may influence health by increasing consumption due to greater cumulative access to unhealthy products.^9,84^ Corporations in UCIs use *co-option of civil society* groups, such as consumer and patient groups, to deflect criticism from public health advocates and confers legitimacy to UCI claims.^17,36,37,41,45,46^ *Manipulation of media* through the exertion of economic influence is a powerful tool corporations in UCIs to shape consumer preferences and discourse around public health initiatives to reduce harm from unhealthy products. Corporations can do this through use of advertising dollars to control content of the media or owning media organisations themselves.^17,41,46,55^ This can be supplemented by campaigns from public relations companies which target the media, legislators and consumers to undermine public health credibility.^17,47,86^

 Corporations in UCIs also enlist *key opinion leaders* and funding health organisations to promote commercial interests in the background of accepted issues and drive accepting of unhealthy products.^17,36,40,45,47,86^ *Manufacturing doubt* refers to casting doubt on scientific evidence documenting negative effects associated with them or focusing on complexities and discrediting scientists who produce such evidence.^6^ This influences health by focusing on ambiguity and lack of consensus and thereby inhibiting regulatory action.^17^ Corporations in UCIs also *frame public health issues* in terms of personal responsibility for making informed choices which takes onus away from practices of that make products more harmful, increase availability and shape the public health narrative.^17,40,45,46,48^

 Corporations in UCIs are also able to build support for industry-friendly stances by *leveraging business affiliations *within an industry or with allied industries with can oppose public health measures.^29,37,40,45,46,47,48,86^ Within this group of practices corporate social responsibility, marketing and advertising and manufacturing doubt received greater attention, all appearing in more than ten out of fourteen frameworks.

 Potential indicators for monitoring UCIs in the domain of preference shaping included the number of events or campaigns aimed at promoting corporate social responsibility^94^; total spending of corporations on marketing with breakdown by advertising medium (eg, print, social media, television)^95^; pricing trends of selected unhealthy products and number of corporate strategies targeting new and vulnerable populations.^9,96,97^ Sources of data for these indicators were varied and included a significant amount of information from corporate documents and resources.^35^ There is extensive monitoring already occurring in this area, including in multi-lateral agencies such as the WHO. The WHO European Office for the Prevention and Control of Noncommunicable Diseases has developed a tool to support Member States in monitoring digital marketing of unhealthy products to children.^98^ This describes how to assess the digital ecosystem within a country and how to collect data of children’s exposure to marketing campaigns for unhealthy products.^98^ Preliminary results of a Norwegian pilot study shows this is a promising tool in demonstrating extent of exposure of children to marketing of unhealthy products, however a larger sample size may be indicated for use to inform policy decision-making, which could also apply for a national government surveillance system.^99^ Allen et al also have developed a Corporate Financial Influence Index using publicly available data, the results of which would be used to monitor UCI pressure on national governments by industry.^100^

###  Knowledge Environment

*Funding research and scientific institutes* sets the agenda for design and analysis methodology and enables data ownership. This can result in biased findings and selective reporting which skews the literature towards industry interests.^17^ Corporations have also *funded researchers to promote industry friendly messaging*, for example Coca Cola company’s Global Energy Balance Network said to have been created to use scientists to downplay the links between obesity and sugary drinks.^101^ *Industry funding of education* through symposia, hospital lectures and public information materials creates educational content that is shaped to favour certain products and procedures over others, typically biased towards industry interests.^87^ Through *establishing scientific advisory boards or institutes* corporations in UCIs are able to control the scientific perception of products once negative impacts are known. These structures are typically staffed with ‘industry friendly’ scientists who support industry position in policy submissions, litigation and when engaging the general public.^17,40,46,47^ Corporations in UCIs can also *control reporting of unfavourable scientific outcomes* by suppressing publication of results that do not suit with the industry narrative.^36,45^ Within this group of practices, funding of research and academic institutions and industry-sponsored education appeared to be prioritised for monitoring, appearing in nine and seven frameworks, respectively.

 A range of potential indicators within the domain of knowledge environment were identified as listed in Table S1 in Supplementary file 1. These included the number of scientists/scientific institutions receiving funding from UCIs,^102^ the number of industry funded education programs^103^ and number of media releases or reports from corporations in UCIs which refuted accepted evidence-based information.^104^ A number of potential data sources were listed primarily focusing on academic literature, internal documents and communication, and institute websites.^40,61,105^ An example of existing work in this area is the US Right to Know group who produce research and journalism relating to the influence of food and chemical corporations.^106^ This civil-society organisation regularly publishes reports of industry attempts to supress research and links to funding of research institutes by corporation. It has been successful in highlighting the prevalence of these corporate practices across both scientific and mainstream media.^107,108^

###  Legal Environment

 Corporations protect themselves against accountability for health impacts by *changing and reinterpreting the law*. By averting liability, corporations in UCIs avoid negative associations with their brand, reparations and regulation of unhealthy products.^17,81^ *Litigation, or the threat of litigation*, is used to deter action that may bring the public’s attention to unhealthy products and practices.^101^ This may be especially relevant to LMICs with fewer resources to fight litigation that is often costly and time-consuming.^29,52^

*Unregulated activity/ externalised costs* refers to corporations keeping prices of harmful products artificially low to encourage consumption. The final price therefore does not reflect the full cost of production meaning that environmental and occupational health costs are passed onto taxpayers.^17,42,50^ Corporations in UCIs can also usurp public health initiatives by using *international activities to avoid domestic regulation*, such as marketing regulations and taxation.^45,46,50^ Within legal environment, litigation appeared to be more of a priority for monitoring than other practices, appearing in nine frameworks as compared to four, two and three for liability, unregulated activity/externalised costs and use of international activities, respectively.

 Potential indicators for monitoring UCI practices in the legal environment included the number of lawsuits related to public health policies or against public health advocates, and limitations of shareholder liability for corporate practices impacting health. Suggested data sources included submissions to the Office of Laws,^109^ transcripts from public hearings,^110^ documents received by governments in which corporations in UCIs threaten legal action,^111^ and media related to law suits.^112^ Existing work in this area includes The African Centre for Tobacco Industry Monitoring & Policy Research which creates country reports for Nigeria and South Africa including data regarding litigation or threat of litigation.^113^ This details lawsuits that have occurred since the last reporting period, including which corporation, the relating policy and the outcome.^113^

###  Extra-Legal Environment

 Corporations may engage in *illegal activities *such as bribing, smuggling, illicit trade and price fixing and often avert criminal prosecution. This undue corporate influence impacts health by circumventing regulatory mechanisms.^17,90^ Furthermore, *bullying and harassment* of government officials, civil society leaders and academics supresses dialogue about harmful impacts of unhealthy commodities and perpetuates the prioritisation of corporate interests in policy and research.^29^ *Opposition fragmentation* refers to practices employed by corporations in UCIs to counteract criticism of products or practices. This is achieved by criticising or discrediting public health advocates, creating multiple voices against public health measures by establishing fake grassroots organisations, also known as astroturfing, and infiltrating or monitoring public health groups.^17,45,46,47,55^ Similar to tax avoidance, *tax evasion* reduces amounts governments have to invest in health promoting infrastructure and services. Fines or prosecution may still be financially attractive to corporations in UCIs where penalties account for a small amount of annual income.^17,29^ Within the extra-legal environment practices, corporate illegal activity appeared in eight frameworks while and harassment, opposition fragmentation and tax evasion appeared in six, five and two frameworks, respectively.

 A number of potential indicators were identified for surveillance of UCI practices within the extra-legal environment. These included the number of reports or prosecutions relating to bribery,^114^ smuggling,^2^ illicit trade^115^ and number of whistle-blower reports of harassment as an industry strategy.^116,117^ A broad range of potential data sources were suggested comprising of whistle-blower reports, investigative journalism pieces, company documents, government reports, and court sentencing documents. One surveillance mechanism developed by Tobacco Tactics from the University of Bath includes illicit tobacco trade in their monitoring program.^118^ Their website contains descriptive reports of illegal trade and smuggling activities from media, government and leaked company documents, as well as a whistle blowing function where people can report directly to the website.

###  Outcomes of Unhealthy Commodity Corporate Practices

 Population and environmental outcomes from the impact of unhealthy commodity corporate practices were identified as important to public health surveillance of UCI corporate practices. Within the population this included consumption patterns, incidence and prevalence of diseases related to the consumption of unhealthy commodity, health and unhealthy product literacy and occupational health of employees.^9,17,18,29,42,45^ The environmental impact identified was the impact on natural systems that affect health.^9,29^ Consumption patterns was identified as an impact in most papers, however overall the identified frameworks did not focus on which impacts should be included for monitoring.^9,18,29,42^ A range of potential indicators and data sources for monitoring health impacts of UCIs were identified in the review. These included level and trends of consumption of unhealthy commodities,^9^ morbidity and mortality of NCD burden attributed to consumption of unhealthy commodities,^119^ level of knowledge of health impacts of unhealthy products and pollution levels released by corporate practices.^9^ Many of the potential data sources listed are already used to undertake monitoring for the relevant indicators, demonstrating the possibility of utilising existing surveillance systems.^18^ For example, the Global Burden of Disease Study examines the morbidity and mortality attributed to dietary risk factors, alcohol and tobacco for each country.^119^ This data could be assessed against the prevalence of corporate practices in a monitoring system.

###  Actors Important for Monitoring of Unhealthy Commodity Corporate Practices

 The literature also emphasized the importance of identifying key individual and institutional actors in the monitoring process, including the government, corporations and industry associated organisations. Within the government, key structures identified were ministries or departments of health, trade and taxation.^13,48,60^ The ministry or department of justice was also seen as important to enforce legislation to control the practices of the UCI.^9,29,86,120^ When considering commercial actors, the literature highlights that corporations are heterogenous and differ greatly in size, resources, production and values, and that this should be considered when selecting a corporation to monitor.^121^ In particular, the power dynamics between corporations and other actors were emphasized as an important factor for surveillance. Size of corporation, location of head office, market share and number of product sales were all identified as important indicators of corporate power.^17,29,122^ For example, in their proposed system for monitoring private-sector organisation within the food industry, Sacks et al suggest monitoring those corporations that have the ‘most’ influence on public health nutrition.^37^ They suggest identifying these organisations through analysis of sales volume or market share by industry type and identifying 15-25 organisations of interest.^37^ The authors also recommend taking into account the size of the organisation, the products and services they provide and the level of influence.^37^ Finally, industry-affiliated organisations were also identified as important actors to including in a monitoring system due to their ability to perpetuate the influence of corporations.^29,37,121,123^ These included peak-representative bodies, corporate subsidiaries, product distributors, industry sponsored research institutes and third-party front groups.^36^

## Discussion

 We conducted a scoping review and synthesis of frameworks that identify corporate strategies and practices across a range of industries and propose a synthesised framework for government-led monitoring of their impact on population health. The review identified 37 corporate practices across a broad range of frameworks which were often siloed by industry (eg, ultra-processed food only), practice (eg, corporate political activity only) or, in the case of Baum et al type of corporation (ie, transnational only). Many corporate practices were named within multiple frameworks, likely indicating a mix of greater visibility and perceived importance. However, since no explicit or relative justification for inclusion of certain corporate practices were made in any framework, is not currently clear why some corporate practices should be prioritised for monitoring over others in any given setting. This finding deserves further research, for example via focused interviews or Delphi studies with key stakeholders in order to understand preferences and rank practices by importance and feasibility, or via analysis which seeks to assign the attributable fraction of burden of disease to specific practice. For each of the 37 corporate practices, we identified a variety of potential indicators and data sources that could be used for surveillance (Table S1, Supplementary file 1). This non-exhaustive list can act to guide to national government policy-makers in their efforts to translate, curate and refine a list of context-appropriate indicators for implementation.We note that the complexity and omnipresent nature of corporations in the UCI mean that no single indicator is sufficient to demonstrate problematic practice. Rather, this framework seeks to identify indicators whose cumulative measurement could help to trigger recognition or flag discussion.

 Synthesis of the corporate strategies identified in different industries has the potential to assist policy-makers by furthering the understanding of how different corporations use the same methods, supporting critical policy reform and directing future research.

 This review provides of the basis for such efforts, demonstrating clear commonalities between the strategies of UCIs across multiple industries, as well as consistent evidence of business links between industries that imply the need for a unified monitoring approach.^10,11,122,124^ However, even frameworks that looked across industries and types of practices included, at most, 26 of the total 37 corporate practices identified. To address the increasing reach and impact of UCI, therefore, findings from this review suggest a need for governments to use frameworks building out from, but more comprehensive than those currently available.^2,22-27^

**Figure 2:**
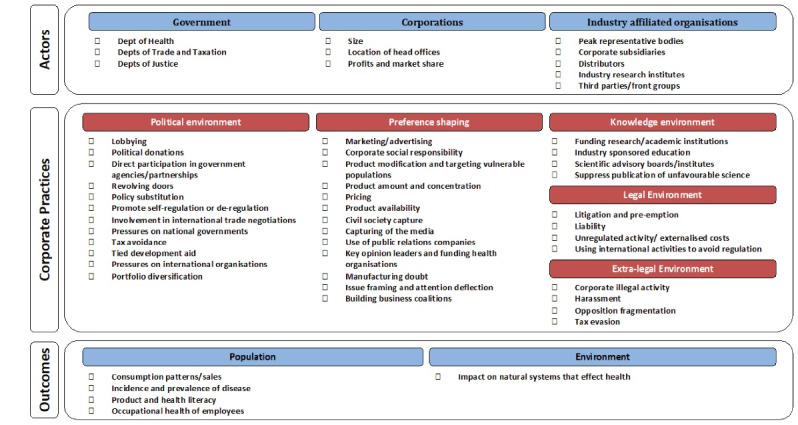
Summary Framework of Major Actors, Corporate Practices and Outcomes.

Figure 2 presents a synthesised framework that identifies key actors, corporate practices and outcomes that should be considered for monitoring of corporations in key UCIs in any given setting. The framework additionally helps to support the re-framing of UCIs as public health threats, similar to other chemical and biologic pollutants, by enabling monitoring that could strengthen casual links between corporate practices and health.^125^

 We present this framework to support the development of national government monitoring of corporations in UCI impact on health, recognizing there is ongoing discussion in three key domains. The first is *who should ‘own’ monitoring* of the health impacts of UCIs. To date monitoring of the health impacts of UCI activities has mainly been the task of civil society and academia, who have been critical in shedding light on the practices of these industries. The findings of this review demonstrate that although a wide range of institutions currently monitor the impact of UCIs’ corporate practices, these efforts are disparate with no identified initiatives monitoring a complete series of practices over multiple UCIs despite the need for a cross-industry approach identified in the literature.^11^ Given the central role of governments in the protection of citizen health, we argue it is necessary to transition to national (and potentially sub-national) governments who bear responsibility for public health monitoring in all other domains, and who hold unique authority to shape the regulatory environment in which corporations in UCIs operate.^35,74^ While, civil society and academic must remain critical partners of such efforts, and are essential for holding public and private actors accountable and monitoring the potential for ‘state capture,’ it is government that has most influence over policy and regulatory settings,^35,74^ for example, by requiring the private sector to disclose certain information which is essential to monitoring.^37,126^

 Given the current paucity of government-led public health surveillance of UCI corporate activities, we acknowledge that government ownership of such a surveillance effort represents a significant step-change for many, if not most governments. This is particularly true for LMIC governments where resources, institutional structures and capacity for public health surveillance are often less well developed than their high-income-country counterparts. Such an endeavour is likely to require cross-disciplinary work, prioritisation of practices and innovation due to the reach of corporate practices into areas outside of national departments of health’s traditional remit.^35,49^ However, given the number, reach and power of corporations, there has never been a more critical time to develop effective mechanisms to improve accountability of these private sector entities for their public health impacts.^127,128^

 Further questions arise regarding where within the administrative infrastructure of the public sector responsibility for corporations in UCI monitoring should lie. While this is ultimately a decision for each jurisdiction, we make the following observations. In line with other public health surveillance, monitoring of UCI corporate activities could be led by health departments but in close collaboration with other government work units due to the reach of corporate practices beyond the remit of health.^35^ Regulatory instruments pertinent to responding to UCI corporate practices, for example, may be found in agriculture, finance, trade and taxation.^49^ Opportunities for an intersectoral approach to regulation of UCIs have been identified in the literature, including the pooling of resources across government departments, and implementing cross-sectoral communication to expand the types of information available beyond on the traditional economic indicators of production or sales.^35^ Global collaboration to create international standards, data collection processes and networks, such as we have seen with the Framework Convention on Tobacco Control, is likely to enable more comprehensive monitoring, particularly with many corporations in UCIs operating across national boundaries.^65^

 In the immediate term, national governments are unlikely to have sufficient resources to monitor all the listed corporate strategies operating within their country. A second decision for national governments to address, therefore, is *which organisations and corporate practices* should be prioritised for monitoring. Criteria to assist with prioritisation may include size of impact on health, scope or prevalence of the corporation or practice within the country, feasibility of monitoring given available resources, and actionability given available policy levers. Further research should be done to assess the application of these different criteria to assist national governments in prioritisation, and evaluate how they may differ by setting. Research to consider how existing evidence and monitoring of non-governmental and academic organisations can be brought together to avoid duplication of efforts is also warranted.

 The third critical decision for national governments is *how to monitor the impact of corporate practices.* Innovation will be required for successful implementation of national government surveillance of corporations in UCIs, both in terms of data collection methods and methods to link corporate practices to health outcomes. This review identified a number of emerging methodologies and technologies that would likely be of use for any scaled-up national surveillance effort. Costa et al, for example, completed an automated content analysis of submissions to the European Union Tobacco Products Directive legislation using text mining.^129^ This quantified the change in proposed legislation related to tobacco industry submissions and demonstrated that tobacco industry lobbying was associated with a significant policy shift towards industry interests. Other novel techniques being employed for assessing corporate practices are sentiment analysis of media, such as Twitter, to analyse corporate responses to policies and artificial intelligence to monitor digital marketing of unhealthy products to children.^130,131^ Such approaches demonstrate the potential for large-scale monitoring of an array of data sources despite limited data collection methods identified in existing frameworks.

 Additionally, while it is currently challenging to assess the magnitude of the distal effects of corporate actions on the health of populations, novel methods are emerging.^132^ For example, Madureira Lima and Galea developed the Corporate Permeation Index, a composite indicator of the degree to which corporate power is embedded in the social, political and cultural fabric of society which is the quantitative expression of the theoretical framework included in this review.^133^ However, this index does not directly consider the environmental and occupational impacts of corporations in UCIs and evidence shows when corporations succeed in growing markets for their commodities, the occupational and environmental harms can be exacerbated. For example, tobacco smoking leads directly to the emission of 2 600 000 tonnes of carbon dioxide and 5 200 000 tonnes of methane per year and also leads to significant deforestation and waste.^134^ Continued development of the methods of assessing the holistic impact of corporations in UCIs is needed, including the use of novel data sources, and should be a focus of national government’s health research agenda. The identification of the likely barriers and enablers to operationalising monitoring of UCI corporate practices and resulting implications for implementation, through interviews with key stakeholders, should be a subject of future research.

###  Strengths and Weaknesses

 This is the first study to review and synthesise frameworks to monitor unhealthy commodity corporate practices across a range of key industries. The review of literature was conducted according to PRISMA guidelines and a novel summary framework provides a comprehensive view of actors, corporate strategies and population and environmental outcomes. This seeks to answer the call for an integrated, cross-sectoral view of the commercial determinants of health. Furthermore, by proposing uptake of UCI monitoring by national governments, this study aims to bridge the gap between existing research and policy choices.

 This review has a number of limitations. First, while a search of the grey literature was conducted, books and book chapters were not included. Second, as our review only examined frameworks published in English and may have missed non-English language work. Third, this study focused on UCI impacts and indicators from tobacco, alcohol and ultra-processed food industries, however we acknowledge that there are many other industries (eg, pharmaceutical, firearms, social media) that also have the potential to contribute to commercial determinants of health. Our hypothesis is that many of the practices used in these industries are used across all profit-seeking commercial actors, however limitations of this approach are that there could be practices specific to other industries that were missed by focus on these specific industries. We also acknowledge that a full perspective of the public health harms of commercial actors requires going beyond commodities, to consider practices and use of power.^135^ This is particularly true for LMICs where governance practices may not be so well-established. The need for increased research in this area from these regions as is consistent with the broader literature.^33^ Finally, this study proposes a preliminary framework for monitoring UCI practices, however further research is needed to develop context-appropriate indicators and understand the barriers and enablers to implementation of surveillance of corporations in UCIs at a national government level. This research should also endeavor to connect monitoring of UCI corporate practices with appropriate government actions.^74^

## Conclusion

 Systematic monitoring of UCIs by governments and public health policy-makers is likely to enable them to better understand and prevent the negative health impacts of corporate practices. This novel analysis of UCI monitoring frameworks provides a synthesis of the range of practices used by corporations in key UCIs that have the potential to impact health. We argue there is significant precedent for monitoring of these practices and the operationalisation of a UCI monitoring system should be the object of future research, development and implementation. This should include consolidation of existing efforts and a focused analysis of the drivers of perceived importance of different corporate practices.

## Ethical issues

 All research activities carried out as part of this project were approved by the Human Ethics Committee at James Cook University, Australia (Application ID: H7935). No ethical issues were identified.

## Competing interests

 Authors declare that they have no competing interests.

## Disclaimer

 The views expressed in this article are those of the authors.

## Supplementary files

Supplementary file 1 contains Table S1.
