# Supplementary material for: National Public Health Surveillance of Corporations in Key Unhealthy Commodity Industries – A Scoping Review and Framework Synthesis
Source: Int J Health Policy Manag. 2023 Mar 5;12:6876. doi: 10.34172/ijhpm.2023.6876 (PMC10425693; doi:10.34172/ijhpm.2023.6876)
Supplement: Supplementary file 1 — contains Table S1. [file ijhpm-12-6876-s001.pdf]

Article title: Systems National Public Health Surveillance of Corporations in Key Unhealthy Commodity Industries – A Scoping Review and Framework Synthesis

Journal name: International Journal of Health Policy and Management (IJHPM)

Authors' information: Elizabeth Bennett[1]*, Stephanie M. Topp[1], Alan Rob Moodie[2]

[1]College of Public Health, Medical and Veterinary Sciences, James Cook University, Townsville, VIC, Australia.

[2]Melbourne School of Population and Global Health, University of Melbourne, Melbourne, VIC, Australia.

(*Corresponding author: dr.elizabethbennett@gmail.com**)**

Supplementary file 1.

Table 3. Potential surveillance indicators for corporate practices and proposed data sources

Practice	Indicator	Purpose	Rationale	Potential data sources	Existing organisational work
**Political Environment**					
**Lobbying**	Number of direct and third-party lobbyists registered from unhealthy commodity industries (UCIs)	To measure the extent of access of UCI representatives to policymakers	Presence of UCI registered lobbyists and a higher number meetings may indicate a greater ability to influence policy makers through direct and indirect access and activity[17,74]	Requests - direct/FOI for politicians' calendar data, minutes and other reports of these meetings, correspondence (e.g. emails) between representatives and the government, whistle blower reports	Corporate Europe Observatory, Curtin University Public Health Advocacy Institute INFORMAS
	Number of documented meetings with UCIs lobbyists reported by politicians	To measure frequency of activity of registered lobbyists via contact with policymakers by, or on behalf of, the industry			
**Political donations**	Number and amount of donations or gifts made to political parties by unhealthy commodity industries	To measure the frequency and extent of donations by UCI corporations, including temporal relationship to policies and campaigns and which political party	Donations and gifts have been noted to have observable effects on health policy in favour of industry. [75-77]A higher number and absolute amount of UCI donations may indicate greater economic influence of UCI and higher potential for favourable decision-making and political agenda-setting because reciprocity may be expected once the party or candidates are in office.[17]	Government/NGO records of donations/gifts, government emails (direct/FOI), media around gifts that are unreported, corporate emails	FCTC, Transparency International
**Direct participation in government**	Number of instances of participation in formal policy processes, such as consultations	To measure the frequency and extent of participation	UCI direct access and involvement in policy making has a demonstratable impact on	Submissions to public consultations, websites of government agencies,	FCTC, Conflicts of Interest Coalition

**agencies, partnerships**		in formal policy-making processes	policy-making with evidence of shifts in policy that are favourable to industry. [17,58,74] Instances and frequency of participation or representation in formal policy-making processes is likely to increase the ability to shift policy in favour of industry interests.	corporate websites, media	
	Number UCI representatives on national policy committees/fora	To measure access to formal policy-making processes			
**Revolving doors**	Number of instances where high-level government officials are employed by corporations within 5 years of government employment and vice versa	To measure the frequency of individuals moving from the public and private sector	Revolving doors enables the industry to acquire inside information on how policy works and gain privileged access to policy fora; conversely, private actors are recruited into public service posts and can then help make public policy. [11,78] Frequency of movement of individuals between the public and private sector flags the ability and potential reach of UCI to influence policy-making processes	Corporation bulletins, business/economic media (e.g. The Economist), LinkedIn/Viadeo and other professional social media sites	Transparency International (NGO), Corporate Europe Observatory
**Involvement in International trade agreements/ negotiations**	Number of trade agreements which favour corporations (e.g. deregulation, removal of barrier to imports and FDI)	To measure the frequency of successful commercial pressure on international trade negotiations and identify which mechanisms are used	Instances of trade agreements which favour UCI are likely to indicate increase availability of unhealthy commodities and decrease price with the potential to lead to higher consumption[61,62]	Government websites, websites of multilateral organisations -World Trade Organisation/ United Nations Industrial Development organisation, websites of international tobacco/food/alcohol trade associations	School of Regulation and Global Governance (RegNet), Australian National University
**Policy substitution**	Number of total policy submissions on non-communicable disease policy made by corporations and associated policy shifts	To measure the extent and impact of policy substitution practices	There is evidence that UCI submissions to policy have a measurable impact on policy.[44,48,49,60] A higher number of submissions is likely to increase the potential for alternate policy to be substituted in the place of evidence-based public health initiatives.	Submissions to public consultations, websites of government agencies, corporate websites, media	

			Measuring the policy shifts associated with these submissions may flag where additional mechanisms to prevent policy substitution are required.[79]		
**Promote self-regulation or de-regulation**	Number of policy submissions on non-communicable disease policy made by corporations which mention self-regulation or deregulation and associated policy shifts that favour UCI	To measure the frequency and impact of UCI promotion of self-regulation or de-regulation	Corporations promote self-regulation or deregulation to avoid legislative interventions.[44,48,49,51] Increased frequency of policy submissions on self-regulation or deregulation could indicate efforts to increase influence over the narrative and public perceptions about the role that governments should play in public health. Measuring the associated policy shifts of these submissions will highlight where additional mechanisms may need to be implemented[79]	Submissions to public consultations, websites of government agencies, corporate websites, media	
**Pressures on national governments**	Degree of market concentration (sum of market share) of corporations in unhealthy commodity industries in the national economy	To measure the extent of structural power UCI have relative to national governments	High market concentration may suggest increased structural power of large UCI corporations compared to national governments and a higher potential influence health policy and regulation[44]	WHO NCD Progress monitor Herfindahl-Hirschman market concentration Index by country	
**Tax avoidance**	Amount of tax being paid by corporations compared to nominal expectation according to local taxation law	To provide a measure the extent of tax avoidance and estimate amount of reduced public tax revenue	Instances and frequency of tax avoidance can lead to reduced public tax revenue with implications for reduced health and social infrastructure and services. [48,63] Quantifying the extent of this reduction may incentivise additional regulatory mechanisms to be implemented to prevent tax avoidance.	Annual reports of corporations, government information on tax rates,	OECD BEPS
	Number of media articles or independent reviews flagging tax avoidance for a corporation	To measure frequency of reporting/documentation of tax avoidance (this indicator is complementary to the one above, as it is assumed relevant financial information may not always be publicly available)			

**Tied Development Aid**	Number and size of international aid programs of corporations with restrictions on purchases/procurement	To measure the frequency and extent of tied development aid	Tied international aid programs have been used to increased influence of UCI in developing countries through de facto export promotion and increased prices of commodities[17]. The presence and scale of such programs may flag where UCI are seeking more influence.		Untying Aid OECD Development Assistance Committee
**Pressures on International Organisations**	Amount of financial support provided annual to key international organisations (e.g. WHO, WTO), absolute and relative to total	To measure the extent of UCI funding of international organisations as a proportion to total funding received	An increased amount of UCI funding of international organisations suggests a greater dependence of international organisations on UCI financial support for operations and increased potential to economically influence health policy-making[17,48]	Information from corporate websites or reports regarding support of international organisations or attendance at meetings, financial reports of key international organisations, visitor registers and participant lists from key events	Corporate Europe Observatory
	Number of corporate delegates to international organisation meetings	To measure the extent of UCI access to International Organisations	UCI direct access to policy-makers has a demonstratable impact on policy-making with evidence of shifts in policy that are favourable to industry. [17,58,74] A higher number of instances of participation or representation in international organisation meetings is likely to increase the ability to shift policy in favour of industry interests.		
**Portfolio diversification**	Number of new industries a corporation is operating in annually (e.g. a tobacco company entering food, alcohol or clothing businesses)	To measure the extent of portfolio diversification of UCI corporations via all mechanisms	Portfolio diversification may be used to protect corporations economically and increase their structural power to influence policy or regulation.[48] Corporations operating in increased number of new industries may have the potential to greater economically influence health policy.	Corporation bulletins, business/economic media (e.g. The Economist), financial reports	Euromonitor
	Number of mergers and acquisitions in new industries annually	To measure the extent of portfolio diversification of UCI corporations via mergers and acquisitions			
**Preference shaping**					

**Corporate social responsibility (CSR)**	Number of events/campaigns aimed at promoting corporate social responsibility	To measure the frequency and extent of UCI CSR	Increased CSR can influence health by increasing exposure to unhealthy products that would otherwise be more tightly regulated and promote product acceptability through association with the image of social commitment.[80-82] CSR has been also been linked to higher per capita of unhealthy products. [83] Presence at one or more UCI events, campaigns and sponsorship funding could indicate an increased potential for UCI to influence attitudes and consumption behaviours.	Information from corporate websites regarding CSR initiatives/philanthropy, websites of major sporting/cultural events regarding funding, information from websites of third-party organisations who receive funding from UCIs, media around events/new products	
	Amount of sponsorship money of sporting or cultural associations/clubs, events/donations/charity affiliations	To quantify the financial extent of UCI CSR and implied economic influence in societies and communities			
**Product modification and targeting vulnerable populations**	Number of strategies to target new/vulnerable markets, and identification of which groups these are targeting (e.g. youth, women)	To measure the extent that new/vulnerable markets are targeted and which groups are at risk of greater exposure to unhealthy products	Higher number of strategies to target new or vulnerable markets could result in increased consumption of unhealthy commodities in certain populations. [84] Knowing which populations are being targeted could inform public health regulation and mechanisms to protect vulnerable groups	Information from company financial reports, shareholder meeting minutes, leaked corporate documents, whistle-blower reports, financial magazines	
	Number of strategies identified to modify products to increase sales/profits of unhealthy commodities	To measure the extent/frequency that product modification is used to increase sales of products, and which products are being modified	Higher number of strategies to modify products could result in increased consumption of unhealthy commodities.[85] Knowing which products are being modified could inform regulation to prevent changes to consumption behaviours		
**Marketing and advertising**	Total overall marketing spends and break-down by medium, timing, target population and content	To measure the extent of marketing to different populations across different mediums and timeframes.	There is evidence that marketing influences behaviours, including purchasing and consumption. [17,86] Increased marketing spends are likely to indicate increased exposure and influence	Company marketing strategy documents, requests to major TV networks, requests to the advertising regulation boards, information from advertising agencies with UCIs as major clients,	WHO - monitoring of junk food advertising in certain regions/countries<br>Truth initiative and Tobacco Documents Legacy library<br>AIMME – NGOS monitoring alcohol marketing in Europe
	Number of violations of marketing codes/regulation	To measure the extent UCI corporations circumvent	Presence and frequency of violations of marketing		

		regulation to limit exposure to UCI marketing	codes/regulations could suggest increased exposure to populations that public health regulation seek to protect (e.g. children). It may also provide insight into the effectiveness of regulation and could inform where changes or alternative mechanisms should be employed to reduce exposure to marketing	information from for profit and academic research groups<br>WHO Euro – CLICK framework for monitoring digital marketing, data mining, mapping paid for exposure<br>Advertising censuses<br>Australian Food and Grocery Council	Canadian government for tobacco<br>Brazilian Code of Marketing of Infant and Toddlers Food and Childcare-related products – census
**Pricing**	Pricing trends of key unhealthy commodity products	To measure changes in prices of unhealthy commodities over time	Lower prices are likely to enable the sale of greater quantities of unhealthy commodities and increase availability to lower income groups [17,87] Decreasing price of commodities overtime could indicate changes to consumption behaviours	Corporate sales documentation, end of year financial reports, Government regulatory documents on minimum prices per unit, taxation, subsidies, corporate websites, media around regulatory actions	Scotland and Australian governments for alcohol
	Change in consumption behaviours as a results of pricing regulations e.g. taxation of goods, government subsidies on products	To measure changes in consumer behaviour as a result of pricing regulation	Pricing regulation is effective in reducing consumption of unhealthy products. [88] High quality and consistent data that demonstrates the impact of pricing regulation may generate support for broader uptake of this regulatory mechanism		
**Product availab lity**	Number of retail units and location within local, state, national jurisdictions e.g.<br>- supermarkets<br>vending machines<br>- fast food outlets<br>- tobacco retailers<br>- bars and alcohol retailers	To measure and map physical access to unhealthy commodities in different regions	A higher number of physical or online locations where the product is available may influence health by increasing consumption due to greater cumulative access to unhealthy products[9,89,90]	Registration documents for re-sellers for unhealthy commodity products, information from corporate websites or industry associations about number of franchises/outlets, Corporate or industry documents regarding reach and purchasing information from	
	Online access to products via participation/purchase via apps/internet (e.g. UberEATS, Amazon, online alcohol delivery)	To measure online access to unhealthy commodities in different regions			
**Product amount and concentration**	Concentrations of harmful ingredients in key unhealthy commodities (e.g. tobacco,	To measure the concentrations of disease-inducing ingredients of	Increased levels of harmful ingredients may indicate the capacity to cause higher levels of	Industry documents/reporting	National governments - Australia, Canada, Brazil, UK, US already do for tobacco

	trans/saturated fats, sugar, ethanol)	unhealthy commodities overtime	disease without changing consumption patterns. Increased portion sizes may influence health by increasing the unhealthy substances are available for consumption in the same serving. [9,91]	Government/ NGO reporting	George Institute for Global Health
	Portion size of product compared to recommended domestic guidelines for consumption and trends in portion size over time	To measure the change in portion size overtime relative to recommended dietary or public health guidelines			
**Civil society capture**	Number of front-groups identified as having connection to industry	To measure extent of front groups used by UCI (where publicly available)	Presence and frequency of front groups or higher report of UCI interference with civil society are likely to influence health by influencing societal preference-shaping behaviour and could suggest increased discursive power of UCI corporations[17,38,40,44,48,49]	Civil society websites, media and reports, consumer watch-dog reports, investigative journalism pieces, FOI/leaked company documents	Corporate Health Observatory
	Number of reports from civil society referring to corporate interference	To measure extent of UCI interference with civil society (used to complement above metric, given it is assumed there will be limits to publicly available information)			
**Capturing of the media**	Number of media organisations owned by UCIs	To measure the extent of UCI media ownership	Frequency of media organisation ownership or increased marketing spend flag the ability of UCI to exert economic influence over media to shape consumer preferences and discourse around unhealthy products.[17,44,49,60]	Company marketing strategy documents, annual reports and financial documents of medica organisations	
	Total overall marketing spends and break-down by medium, timing, target population and content, relative to total marketing revenue	To measure the proportion of marketing revenue from UCI and implied economic influence of UCI over media organisations			
**Use of Public relations companies**	Number of public relations companies identifying corporations as clients	To measure the extent that UCI corporations use public relations companies for campaigns	Frequency of use of PR companies by UCI corporations may indicate their efforts to increase influence over narratives around unhealthy products with the media, legislators and consumers.[53,91,92]		
**Key opinion leaders and funding health organisations**	Number of paid opinion leaders and health organisations receiving funding from UCI	To measure the frequency of payments to opinion leaders/health organisations and implied economic influence (where publicly available)	Frequency and scale of UCI funding of opinion leaders or health organisations may flag increased economic influence and potential for increased promotion of commercial		

			interests by driving acceptance of unhealthy products. [38,43,48,53,91,92]		
**Manufacturing doubt**	Number of media releases/reports from UCIs framing issue contrary to scientific evidence	To measure the frequency of UCI casting doubt on scientific evidence or on the scientific community who produce such evidence	Instances of manufacturing doubt indicate the potential for increased discursive power of UCI corporations and the increased potential to influence health via lack of consensus and inhibition of regulatory action.[17]	Submissions to public consultations, websites of government agencies, corporate websites, media	SEATCA
**Issue framing and attention deflection**	Number of policy submissions promotion personal responsibility for healthy consumption from UCI	To measure the incidence of issue-framing towards personal responsibility in policy-making processes	Increased incidence of issue-framing towards personal responsibility for making informed choices could suggest a greater potential for UCI to shape public narrative on the role for public health regulation in preventing harm from unhealthy products [43,48,49,51,92]	Corporation Twitter + other social media Industry reports and media releases	
**Building business coalitions**	Number of business coalitions by industry	To measure the number of business coalitions in different UCI	An increased number of UCI business coalitions, and higher activity in the policy-making process of these coalitions, could indicate an increased ability to influence to oppose public health measures [29,40,43,48,49,51,53,91]	Business coalition websites, submissions to public consultations, websites of government agencies, corporate websites, media	
	Number of policy submissions from identified business coalitions relating to public-health regulation	To measure the involvement of UCI business coalitions in the policy-making process			
**Knowledge environment**					
**Funding research/ institutions**	Number of scientists/scientific institutions receiving funding from UCIs	To measure the incidence of UCI funding scientific research (where publicly available)	An increased number of scientists/scientific institutions/publications receiving UCI funding could indicate increased potential for biased findings and selective reporting which skews the literature towards industry interests[17]	Websites of scientific institutions, data from peer reviewed journals on conflicts of interest	US Right to Know
	Number of papers published in peer-reviewed journals with UCIs as documented funders	To measure the incidence of UCI funding scientific research (used to complement above metric, as all funding may not be publicly reported)			
**Industry sponsored education**	Number of conferences/prizes/education material paid by UCIs<br>Number of industry funded education programs (e.g. DrinkWise)	To measure the prevalence of UCI funding of educational programs and events	A higher number of UCI sponsored educations indicate increased potential for educational content to be shaped to favour UCI products/procedures.[93]	Corporate website and promotional materials<br>Conference/academic institute website/documents	

**Scientific advisory boards/science institutes**	Number of scientific advisory boards and research institutes funded, owned or established by UCI corporations	To measure the incidence of UCI funded, owned or established scientific organisations	A higher number of UCI established or sponsored scientific advisory boards or institutes could indicate higher levels or reach of UCI influence over scientific outputs, policy submissions, litigation and other public-interest activities, in favour certain products. [17,43,49,53]	Websites of scientific institutions, data from peer reviewed journals on conflicts of interest	
**Suppress publication of unfavourable science**	Number of reports of suppression of publication of unfavourable science	To measure the frequency of suppression of unfavourable science	A greater frequency of suppression of unfavourable science could indicate increased discursive power of UCI and a greater ability to shape the scientific to suit industry interests.[38,48]	Whistle-blower reports, investigative journalism pieces, FOI/leaked company documents,	
**Legal Environment**					
**Litigation**	Number of lawsuits brought by UCI related to public health policies or agencies	To measure the frequency of litigation to undermine public health policies or agencies	Higher frequency of litigation, or the threat of litigation, may indicate an increased likelihood of suppression of policies or practices used to limit consumption of unhealthy commodiities.[94] It may also serve as an indicator of the amount of public resources being used to respond to litigation. [29,57]	Submissions to the office of laws, government documents received by corporations threatening legal action, media around legal battles (or threat of)	The African Centre for Tobacco Industry Monitoring and Policy Research
**Liability**	Number of UCI corporations with limitations of shareholder liability for corporate practices affecting health	To measure the frequency of UCI corporations without liability for detrimental health impacts of their products on health	A higher number of corporations with limitations of liability for corporate practices affecting health could indicate an increased ability of UCI corporations to avoid reparations and regulation of unhealthy products. [17,86]	Corporate registration documents	
**Unregulated activity/externalised costs**	The price difference between current producer prices and true costs[95]	To measure the social and health costs of unhealthy commodity products that are not currently considered in the cost structure of UCI production	A higher price differential between the current producer prices of unhealthy commodities and true costs (e.g. health care spend treating non-communicable diseases related to unhealthy commodity	Euromonitor data on price of products	

			consumption) suggests a greater burden of the social and health costs passed on to broader society. As these external costs are not yet included in market prices, and in the absence of compensation, this leads to welfare losses for society as a whole.[17,45,55]		
**Using international activities to avoid domestic regulation**	Number of reports of use of international activities to avoid domestic regulation	To measure the frequency and modes of UCI use of international activities to avoid domestic regulation	Use of international activities to avoid domestic regulation can indicate potential for increased consumption of unhealthy products by avoiding regulation for practices such as marketing and pricing.[48,49,55] Documentation of modes may assist in strengthened regulatory mechanisms.	Whistle-blower reports, investigative journalism pieces, FOI/leaked company documents, government reports, court sentencing documents	Tobacco Tactics
**Extra-legal environment**					
**Corporate illegal activity**	Number of reports of bribery/smuggling/illicit trade/ price fixing[17]	To measure the incidence of corporate illegal activity	Frequency of corporate illegal activity helps gauge the potential for corporate influence by circumventing regulatory mechanisms. [17,96]	Whistle-blower reports, investigative journalism pieces, FOI/leaked company documents, government reports, court sentencing documents	Tobacco Tactics
**Harassment**	Number of reports of harassment or bullying of researchers or policy-makers by UCI	To measure the prevalence and types of harassment by UCI as a practice or strategy	Instances of harassment by UCI may indicate attempts by UCIs to suppress dialogue about harmful impacts of unhealthy commodities. [29]	Whistle-blower reports, investigative journalism pieces, FOI/leaked company documents,	
	Number of whistle-blower reports of harassment as an UCI strategy				
**Opposition fragmentation**	Number of front groups identified as being infiltrated by UCI corporations	To measure the frequency that front groups are used by UCI and implied creation of multiple voices to support industry interests	Presence and number of front groups linked to UCI and frequency of discrediting of public health organisations provides a measure of ability of UCI to shape the public discourse around unhealthy products or practices.[48,49,53,60,92]	Whistle-blower reports, investigative journalism pieces, FOI/leaked company documents, corporate media (e.g. Twitter, Facebook), corporate websites	
	Number of media reports discrediting public health organisations linked to UCI	To measure the incidence of criticism or discrediting of public health organisations by UCI			

**Tax evasion**	Amount of tax being paid by corporations compared to what is expected	To measure the extent of tax evasion and estimate amount of reduced public tax revenue	Presence of tax evasion flags corporate practices not in line with public interest. Increased tax evasion reduces public tax revenue directly impacting health and social infrastructure and services. [48,63]	Annual reports of corporations, government information on tax rates,	
	Number of media articles or independent reviews flagging tax evasion for a corporation	To measure frequency of tax evasion (to complement above indicators as not all relevant financial information may be publicly reported)			

References:

1. Greenhalgh S. Making China safe for Coke: how Coca-Cola shaped obesity science and policy in China. *BMJ.* 2019;364:k5050. doi:10.1136/bmj.k5050
2. Moodie R, Stuckler D, Monteiro C, et al. Profits and pandemics: prevention of harmful effects of tobacco, alcohol, and ultra-processed food and drink industries. *Lancet.* 2013;381(9867):670-679. doi:10.1016/S0140-6736(12)62089-3
3. Stuckler D, McKee M, Ebrahim S, Basu S. Manufacturing epidemics: the role of global producers in increased consumption of unhealthy commodities including processed foods, alcohol, and tobacco. *PLoS Med.* 2012;9(6):e1001235. doi:10.1371/journal.pmed.1001235
4. Wiist W. *The bottom line or public health: tactics corporations use to influence health and health policy and what we can do to counter them*. Oxford: Oxford University Press; 2010.
5. Freudenberg N. *Lethal but legal: corporations, consumption, and protecting health*. New York: USA Oxford University Press; 2014.
6. Oreskes NC, EM. *Merchants of doubt: how a handful of scientists obscured the truth on issues from tobacco smoke to global warming*. New York: Bloomsbury Press; 2010.
7. Marmot M, Bell R. Social determinants and non-communicable diseases: time for integrated action. *BMJ.* 2019;364:l251. doi:10.1136/bmj.l251
8. Casswell S. Vested interests in addiction research and policy. Why do we not see the corporate interests of the alcohol industry as clearly as we see those of the tobacco industry? *Addiction.* 2013;108(4):680-685. doi:10.1111/add.12011
9. Jahiel RI. Corporation-induced diseases, upstream epidemiologic surveillance, and urban health. *J Urban Health.* 2008;85(4):517-531. doi:10.1007/s11524-008-9283-x
10. Smith K, Dorfman L, Freudenberg N, et al. Tobacco, Alcohol, and Processed Food Industries - Why Do Public Health Practitioners View Them So Differently? *Front Public Health.* 2016;4:64. doi:10.3389/fpubh.2016.00064
11. Knai C, Petticrew M, Capewell S, et al. The case for developing a cohesive systems approach to research across unhealthy commodity industries. *BMJ Global Health.* 2021;6(2):e003543. doi:10.1136/bmjgh-2020-003543
12. Melaku YA, Renzaho A, Gill TK, et al. Burden and trend of diet-related non-communicable diseases in Australia and comparison with 34 OECD countries, 1990-2015: findings from the Global Burden of Disease Study 2015. *Eur J Nutr.* 2019;58(3):1299-1313. doi:10.1007/s00394-018-1656-7
13. World Health Organization. *Fiscal Policies for Diet and Prevention of Noncommunicable Diseases.* Geneva: World Health Organization;2015.
14. Moodie AR. The Relentless Enemies of Science. *Am J Public Health.* 2017;107(7):1016-1017. doi:10.2105/AJPH.2017.303860
15. Lee K, Freudenberg N. Addressing the commercial determinants of health begins with clearer definition and measurement. *Global Health Promotion.* 2020;27(2):3-5. doi:10.1177/1757975920931249

16. Babor TF, Robaina K. Public health, academic medicine, and the alcohol industry's corporate social responsibility activities. *Am J Public Health.* 2013;103(2):206-214. doi:10.2105/AJPH.2012.300847

17. Madureira Lima J, Galea S. Corporate practices and health: a framework and mechanisms. *Global Health.* 2018;14(1):21. doi:10.1186/s12992-018-0336-y

18. McCambridge J, Coleman R, McEachern J. Public Health Surveillance Studies of Alcohol Industry Market and Political Strategies: A Systematic Review. *J Stud Alcohol Drugs.* 2019;80(2):149-157.

19. Babor TF. Editor's Corner: The Role of Public Health Surveillance in Protecting Young People From Alcohol Marketing. *J Stud Alcohol Drugs.* 2016;77(1):5-6.

20. Babor TF, Robaina K, Jernigan D. The influence of industry actions on the availability of alcoholic beverages in the African region. *Addiction.* 2015;110(4):561-571. doi:10.1111/add.12832

21. World Health Organization. *Communicable disease surveillance and response systems: guide to monitoring and evaluation.* Geneva: World Health Organization;2006.

22. Collin J, Hill SE, Kandlik Eltanani M, Plotnikova E, Ralston R, Smith KE. Can public health reconcile profits and pandemics? An analysis of attitudes to commercial sector engagement in health policy and research. *PLoS One.* 2017;12(9):e0182612. doi:10.1371/journal.pone.0182612

23. Anaf J, Baum FE, Fisher M, Harris E, Friel S. Assessing the health impact of transnational corporations: a case study on McDonald's Australia. *Global Health.* 2017;13(1):7. doi:10.1186/s12992-016-0230-4

24. Allen L, Bloomfield A, Working Group of the Global Coordination Mechanism on N. Engaging the private sector to strengthen NCD prevention and control. *Lancet Glob Health.* 2016;4(12):e897-e898. doi:10.1016/S2214-109X(16)30216-9

25. Collins T, Mikkelsen, B., Axelrod, S. Interact, engage or partner? Working with the private sector for the prevention and control of noncommunicable diseases. *Cardiovasc Diagn Ther.* 2018. doi:10.21037/cdt.2018.08.04

26. Buse K, Tanaka S, Hawkes S. Healthy people and healthy profits? Elaborating a conceptual framework for governing the commercial determinants of non-communicable diseases and identifying options for reducing risk exposure. *Global Health.* 2017;13(1):34. doi:10.1186/s12992-017-0255-3

27. Ng S, Kelly B, Yeatman H, Swinburn B, Karupaiah T. Tracking progress from policy development to implementation: A case study on adoption of mandatory regulation for nutrition labelling in malaysia. *Nutrients.* 2021;13(2):1-18. doi:10.3390/nu13020457

28. Lacy-Nichols J, Marten R. Power and the commercial determinants of health: ideas for a research agenda. *BMJ Glob Health.* 2021;6(2). doi:10.1136/bmjgh-2020-003850

29. Baum FE, Sanders DM, Fisher M, et al. Assessing the health impact of transnational corporations: its importance and a framework. *Global Health.* 2016;12(1):27. doi:10.1186/s12992-016-0164-x

30. Babic M, Fichtner J, Heemskerk EM. States versus Corporations: Rethinking the Power of Business in International Politics. *The International Spectator.* 2017;52(4):20-43. doi:10.1080/03932729.2017.1389151

31. Various: Public company profile. 2021. Accessed October 8th, 2021.

32. Revenue, excluding grants (% of GDP). 2021. Accessed 6th October 2021.

33. Moodie R, Bennett E, Kwong EJL, et al. Ultra-Processed Profits: The Political Economy of Countering the Global Spread of Ultra-Processed Foods - A Synthesis Review on the Market and Political Practices of Transnational Food Corporations and Strategic Public Health Responses. *Int J Health Policy Manag.* 2021. doi:10.34172/ijhpm.2021.45

34. Hirschhorn N. Corporate social responsibility and the tobacco industry: hope or hype? *Tob Control.* 2004;13(4):447-453. doi:10.1136/tc.2003.006676

35. Smith K, Dorfman L, Freudenberg N, et al. Tobacco, Alcohol, and Processed Food Industries - Why Do Public Health Practitioners View Them So Differently? *Frontiers in public health.* 2016;4:64-64. doi:10.3389/fpubh.2016.00064

36. <0829573509345481.pdf>.

37. Lencucha R, Thow AM. Intersectoral policy on industries that produce unhealthy commodities: governing in a new era of the global economy? *BMJ GLOBAL HEALTH.* 2020;5(8). doi:10.1136/bmjgh-2019-002246

38. Legg T, Hatchard J, Gilmore AB. The Science for Profit Model-How and why corporations influence science and the use of science in policy and practice. *PLoS One.* 2021;16(6):e0253272. doi:10.1371/journal.pone.0253272

39. Lencucha R, Thow AM. Intersectoral policy on industries that produce unhealthy commodities: governing in a new era of the global economy? *BMJ Glob Health.* 2020;5(8). doi:10.1136/bmjgh-2019-002246

40. Sacks G, Swinburn B, Kraak V, et al. A proposed approach to monitor private-sector policies and practices related to food environments, obesity and non-communicable disease prevention. *Obes Rev.* 2013;14 Suppl 1:38-48. doi:10.1111/obr.12074

41. Ng S, Sacks G, Kelly B, et al. Benchmarking the transparency, comprehensiveness and specificity of population nutrition commitments of major food companies in Malaysia. *Globalization and Health.* 2020;16(1). doi:10.1186/s12992-020-00560-9

42. Swinburn B, Sacks G, Vandevijvere S, et al. INFORMAS (International Network for Food and Obesity/non-communicable diseases Research, Monitoring and Action Support): overview and key principles. *Obes Rev.* 2013;14 Suppl 1:1-12. doi:10.1111/obr.12087

43. Mialon M, Swinburn B, Sacks G. A proposed approach to systematically identify and monitor the corporate political activity of the food industry with respect to public health using publicly available information. *Obes Rev.* 2015;16(7):519-530. doi:10.1111/obr.12289

44. Wood B, Baker P, Sacks G. Conceptualising the Commercial Determinants of Health Using a Power Lens: A Review and Synthesis of Existing Frameworks. *International Journal of Health Policy and Management.* 2021. doi:10.34172/ijhpm.2021.05

45. Wiist WH. Public health and the anticorporate movement: rationale and recommendations. *Am J Public Health.* 2006;96(8):1370-1375. doi:10.2105/AJPH.2005.072298

46. Tricco AC, Lillie E, Zarin W, et al. PRISMA Extension for Scoping Reviews (PRISMA-ScR): Checklist and Explanation. *Ann Intern Med.* 2018;169(7):467-473. doi:10.7326/M18-0850

47. Walt G, Gilson L. Reforming the health sector in developing countries: the central role of policy analysis. *Health Policy Plan.* 1994;9(4):353-370. doi:10.1093/heapol/9.4.353

48. Stillman F, Hoang M, Linton R, Ritthiphakdee B, Trochim W. Mapping tobacco industry strategies in South East Asia for action planning and surveillance. *Tob Control.* 2008;17(1):e1. doi:10.1136/tc.2006.017988

49. Trochim WM, Stillman FA, Clark PI, Schmitt CL. Development of a model of the tobacco industry's interference with tobacco control programmes. *Tob Control.* 2003;12(2):140-147.

50. Ulucanlar S, Fooks GJ, Gilmore AB. The Policy Dystopia Model: An Interpretive Analysis of Tobacco Industry Political Activity. *PLoS medicine.* 2016;13(9):e1002125-e1002125. doi:10.1371/journal.pmed.1002125

51. Knai C, Petticrew, M., Mays, N., Capewell, S., Cassidy, R., Cummins, S., Eastmure, E., Fafard, P., Hawkins, B., Jensin, JD., Katikireddi, S., Mwatsama, M., Orford., J, Weishaar, H. Systems thinking as a framework for analyzing commerical determinants of health. *The Milbank Quarterly.* 2018;96(3):472-498.

52. Keshavarz Mohammadi N, Taheri F, Motallebi M, et al. Development of a national conceptual framework and measuring tool for Organisational Social Responsibility and Accountability for Health (OSRAH). *Global Health Promotion.* 2020;27(2):17-25. doi:10.1177/1757975918789346

53. Ulucanlar S, Fooks GJ, Gilmore AB. The Policy Dystopia Model: An Interpretive Analysis of Tobacco Industry Political Activity. *PLoS Med.* 2016;13(9):e1002125. doi:10.1371/journal.pmed.1002125

54. Keshavarz Mohammadi N, Taheri F, Motallebi M, et al. Development of a national conceptual framework and measuring tool for Organisational Social Responsibility and Accountability for Health (OSRAH). *Global Health Promotion.* 2019;27(2):17-25. doi:10.1177/1757975918789346

55. Baum F, Freeman T, Sanders D, Labonte R, Lawless A, Javanparast S. Comprehensive primary health care under neo-liberalism in Australia. *Soc Sci Med.* 2016;168:43-52. doi:10.1016/j.socscimed.2016.09.005

56. Mindell JS, Reynolds L, Cohen DL, McKee M. All in this together: the corporate capture of public health. *BMJ.* 2012;345:e8082. doi:10.1136/bmj.e8082

57. Wiist WH. Citizens United, public health, and democracy: the Supreme Court ruling, its implications, and proposed action. *Am J Public Health.* 2011;101(7):1172-1179. doi:10.2105/AJPH.2010.300043

58. Jorgensen PD. Pharmaceuticals, political money, and public policy: a theoretical and empirical agenda. *J Law Med Ethics.* 2013;41(3):561-570. doi:10.1111/jlme.12065

59. Nestle M. Influencing government: food lobbies and lobbyists. *Food politics.* Vol 2nd. Berkeley: University of California Press,; 2010:95-111.

60. Mialon M, Swinburn B, Allender S, Sacks G. Systematic examination of publicly-available information reveals the diverse and extensive corporate political activity of the food industry in Australia. *BMC Public Health.* 2016;16:283. doi:10.1186/s12889-016-2955-7

61. Blouin C, Chopra M, van der Hoeven R. Trade and social determinants of health. *Lancet.* 2009;373(9662):502-507. doi:10.1016/S0140-6736(08)61777-8

62. Nestle M, Jacobson MF. Halting the obesity epidemic: a public health policy approach. *Public Health Rep.* 2000;115(1):12-24. doi:10.1093/phr/115.1.12

63. Mialon M. An overview of the commercial determinants of health. *Global Health.* 2020;16(1):74. doi:10.1186/s12992-020-00607-x

64. Scrinis G. Ultra-processed foods and the corporate capture of nutrition—an essay by Gyorgy Scrinis. *BMJ.* 2020;371:m4601. doi:10.1136/bmj.m4601

65. Schram A, Ruckert A, VanDuzer JA, et al. A conceptual framework for investigating the impacts of international trade and investment agreements on noncommunicable disease risk factors. *Health Policy Plan.* 2018;33(1):123-136. doi:10.1093/heapol/czx133

66. Stuckler D, Reeves A, Loopstra R, McKee M. Textual analysis of sugar industry influence on the World Health Organization's 2015 sugars intake guideline. *Bull World Health Organ.* 2016;94(8):566-573. doi:10.2471/BLT.15.165852

67. Givel MS, Glantz SA. Tobacco lobby political influence on US state legislatures in the 1990s. *Tob Control.* 2001;10(2):124-134. doi:10.1136/tc.10.2.124

68. Corporate Europe Observatory. Lobbying the EU. 2020; https://corporateeurope.org/en/lobbying-the-eu. Accessed 08 Mar, 2020.

69. Traynor I. 30,000 lobbyists and counting: is Brussels under corporate sway? *The Guardian*2014.

70. World Health Organization. *WHO framework convention on tobacco control*. Geneva: World Health Organization; 2003.

71. Freeman B, Chapman S. British American tobacco on facebook: undermining Article 13 of the global World Health Organization framework convention on tobacco control. *Tobacco control.* 2010;19(3):e1-e9.

72. Chung-Hall J, Craig L, Gravely S, Sansone N, Fong GT. Impact of the WHO FCTC over the first decade: a global evidence review prepared for the Impact Assessment Expert Group. *Tob Control.* 2019;28(Suppl 2):s119-s128. doi:10.1136/tobaccocontrol-2018-054389

73. Global Tobacco Index. Global Tobacco Index. 2020; https://globaltobaccoindex.org/countries. Accessed Feb 18, 2020.

74. Lauber K, Rippin H, Wickramasinghe K, Gilmore AB. Corporate political activity in the context of sugar-sweetened beverage tax policy in the WHO European Region. *European Journal of Public Health.* 2022:ckac117. doi:10.1093/eurpub/ckac117

75. Fooks GJ, Gilmore AB. Corporate Philanthropy, Political Influence, and Health Policy. *PLOS ONE.* 2013;8(11):e80864. doi:10.1371/journal.pone.0080864

76. Khan MS, Meghani A, Liverani M, Roychowdhury I, Parkhurst J. How do external donors influence national health policy processes? Experiences of domestic policy actors in Cambodia and Pakistan. *Health Policy and Planning.* 2018;33(2):215-223. doi:10.1093/heapol/czx145

77. Tesler LE, Malone RE. Corporate philanthropy, lobbying, and public health policy. *Am J Public Health.* 2008;98(12):2123-2133. doi:10.2105/ajph.2007.128231

78. Miller D, Harkins C. Corporate strategy, corporate capture: Food and alcohol industry lobbying and public health. *Critical Social Policy.* 2010;30(4):564-589. doi:10.1177/0261018310376805

79. Mialon M, Vandevijvere S, Carriedo-Lutzenkirchen A, et al. Mechanisms for addressing and managing the influence of corporations on public health policy, research and practice: A scoping review. *BMJ Open.* 2020;10(7):e034082. doi:http://dx.doi.org/10.1136/bmjopen-2019-034082

80. Sebrie EM, Glantz SA. Attempts to undermine tobacco control: tobacco industry "youth smoking prevention" programs to undermine meaningful tobacco control in Latin America. *Am J Public Health.* 2007;97(8):1357-1367. doi:10.2105/AJPH.2006.094128

81. Wakefield M, Terry-McElrath Y, Emery S, et al. Effect of televised, tobacco company-funded smoking prevention advertising on youth smoking-related beliefs, intentions, and behavior. *Am J Public Health.* 2006;96(12):2154-2160. doi:10.2105/AJPH.2005.083352

82. Brownell KD, Warner KE. The perils of ignoring history: Big Tobacco played dirty and millions died. How similar is Big Food? *Milbank Q.* 2009;87(1):259-294. doi:10.1111/j.1468-0009.2009.00555.x

83. Conde K, Peltzer RI, Pantani D, Pinsky I, Cremonte M. Alcohol industry, corporate social responsibility and country features in Latin America. *Drug and Alcohol Review.* 2021;40(3):423-430. doi:10.1111/dar.13208

84. Esser MB, Jernigan DH. Policy Approaches for Regulating Alcohol Marketing in a Global Context: A Public Health Perspective. *Annu Rev Public Health.* 2018;39:385-401. doi:10.1146/annurev-publhealth-040617-014711

85. Scrinis G, Monteiro CA. Ultra-processed foods and the limits of product reformulation. *Public Health Nutrition.* 2018;21(1):247-252. doi:10.1017/S1368980017001392

86. Brezis M, Wiist WH. Vulnerability of health to market forces. *Med Care.* 2011;49(3):232-239. doi:10.1097/MLR.0b013e31820ab638

87. Kickbusch I, Allen L, Franz C. The commercial determinants of health. *Lancet Glob Health.* 2016;4(12):e895-e896. doi:10.1016/S2214-109X(16)30217-0

88. Passos CMd, Maia EG, Levy RB, Martins APB, Claro RM. Association between the price of ultra-processed foods and obesity in Brazil. *Nutrition, Metabolism and Cardiovascular Diseases.* 2020;30(4):589-598. doi:https://doi.org/10.1016/j.numecd.2019.12.011

89. Bryden A, Roberts B, McKee M, Petticrew M. A systematic review of the influence on alcohol use of community level availability and marketing of alcohol. *Health & Place.* 2012;18(2):349-357. doi:https://doi.org/10.1016/j.healthplace.2011.11.003

90. Miles H, Apeldoorn B, McKerchar C, Curl A, Crossin R. Describing and characterising on-demand delivery of unhealthy commodities in New Zealand. *Australian and New Zealand Journal of Public Health.* 2022;46(4):429-437. doi:https://doi.org/10.1111/1753-6405.13230

91. Jahiel RI, Babor TF. Industrial epidemics, public health advocacy and the alcohol industry: lessons from other fields. *Addiction.* 2007;102(9):1335-1339. doi:10.1111/j.1360-0443.2007.01900.x

92. Madureira Lima J, Galea S. Corporate practices and health: a framework and mechanisms. *Globalization and Health.* 2018;14(1):21. doi:10.1186/s12992-018-0336-y

93. Sah S, Fugh-Berman A. Physicians under the influence: social psychology and industry marketing strategies. *J Law Med Ethics.* 2013;41(3):665-672. doi:10.1111/jlme.12076

94. Ibrahim JK, Glantz SA. Tobacco industry litigation strategies to oppose tobacco control media campaigns. *Tobacco control.* 2006;15(1):50-58. doi:10.1136/tc.2005.014142

95. Pieper M, Michalke A, Gaugler T. Calculation of external climate costs for food highlights inadequate pricing of animal products. *Nature Communications.* 2020;11(1):6117. doi:10.1038/s41467-020-19474-6

96. Joossens L, Merriman D, Ross H, Raw M. The impact of eliminating the global illicit cigarette trade on health and revenue. *Addiction.* 2010;105(9):1640-1649. doi:10.1111/j.1360-0443.2010.03018.x

97. Dahlsrud A. How corporate social responsibility is defined: an analysis of 37 definitions. *Corporate Social Responsibility and Environmental Management.* 2008;15(1):1-13. doi:https://doi.org/10.1002/csr.132

98. da Silva KB, de Oliveira MIC, Boccolini CS, de Oliveira Fonseca Sally E. Illegal commercial promotion of products competing with breastfeeding. *Revista de Saude Publica.* 2020;54. doi:10.11606/S1518-8787.2020054000854

99. Lacy-Nichols J, Scrinis G, Carey R. The evolution of Coca-Cola Australia's soft drink reformulation strategy 2003-2017: A thematic analysis of corporate documents. *FOOD POLICY.* 2020;90. doi:10.1016/j.foodpol.2019.101793

100. Oreskes N. *Merchants of doubt : how a handful of scientists obscured the truth on issues from tobacco smoke to global warming*: 1st U.S. edition. New York : Bloomsbury Press, 2011. ©2010.; 2011.

101. McCreanor T, Greenaway A, Moewaka Barnes H, Borell S, Gregory A. Youth identity formation and contemporary alcohol marketing. *Critical Public Health.* 2005;15(3):251-262. doi:10.1080/09581590500372345

102. Jernigan D, Ross CS. The Alcohol Marketing Landscape: Alcohol Industry Size, Structure, Strategies, and Public Health Responses. *J Stud Alcohol Drugs Suppl.* 2020;Sup 19:13-25.

103. Kim Y. Consumer Responses to the Food Industry's Proactive and Passive Environmental CSR, Factoring in Price as CSR Tradeoff. *Journal of Business Ethics.* 2017;140(2):307-321.

104. Machado PP, Claro RM, Canella DS, Sarti FM, Levy RB. Price and convenience: The influence of supermarkets on consumption of ultra-processed foods and beverages in Brazil. *Appetite.* 2017;116:381-388. doi:10.1016/j.appet.2017.05.027

105. World Health Organisation European Office. *Monitoring and restricting digital marketing,.* Moscow, Russian Federation: WHO;2018.

106. Steinnes KHV. *Mapping the landscape of digital food marketing: Investigating expusure of digital food and drink advertisements to Norwegian children.* Oslo: Oslo Metropolitan University 2020.

107. Allen LN, Wigley S, Holmer H. Assessing the association between Corporate Financial Influence and implementation of policies to tackle commercial determinants of non-communicable diseases: A cross-sectional analysis of 172 countries. *Social Science & Medicine.* 2022;297:114825. doi:https://doi.org/10.1016/j.socscimed.2022.114825

108. Griffin S. Coca-Cola's work with academics was a "low point in history of public health". *BMJ.* 2020;370:m3075. doi:10.1136/bmj.m3075

109. Serodio PM, McKee M, Stuckler D. Coca-Cola - a model of transparency in research partnerships? A network analysis of Coca-Cola's research funding (2008-2016). *Public Health Nutr.* 2018;21(9):1594-1607. doi:10.1017/S136898001700307X

110. Mialon M, McCambridge J. Alcohol industry corporate social responsibility initiatives and harmful drinking: a systematic review. *European Journal of Public Health.* 2018;28(4):664-673. doi:10.1093/eurpub/cky065

111. Farrell J, McConnell K, Brulle R. Evidence-based strategies to combat scientific misinformation. *Nature Climate Change.* 2019;9(3):191-195. doi:10.1038/s41558-018-0368-6

112. Serodio P, Ruskin G, McKee M, Stuckler D. Evaluating Coca-Cola's attempts to influence public health 'in their own words': analysis of Coca-Cola emails with public health academics leading the Global Energy Balance Network. *Public Health Nutrition.* 2020;23(14):2647-2653. doi:10.1017/S1368980020002098

113. U.S Right to Know. U.S Right to Know: Our Investigations. 20210; https://usrtk.org/our-investigations/.

114. Steele S, Ruskin G, Stuckler D. Pushing partnerships: corporate influence on research and policy via the International Life Sciences Institute. *Public Health Nutrition.* 2020;23(11):2032-2040. doi:10.1017/S1368980019005184

115. Jacobs A. A Shadowy Industry Group Shapes Food Policy Around the World. *The New York Times*2019.

116. Greenhalgh E, George A, Zhou S, Slattery, C and Liberman, J. *16.5.1 Litigation in national courts to overturn state or national legislation.* Melbourne: Cancer Council Victoria;2021.

117. Mariath AB, Martins APB. Sugary drinks taxation: industry's lobbying strategies, practices, and arguments in the Brazilian legislature. *Public health nutrition.* 2021:1-30. doi:http://dx.doi.org/10.1017/S136898002100149X

118. Flores ML, Barnoya J, Mejia R, Alderete E, Pérez-Stable EJ. Litigation in Argentina: challenging the tobacco industry. *Tobacco Control.* 2006;15(2):90. doi:10.1136/tc.2004.010835

119. Bhatta DN, Crosbie E, Bialous SA, Glantz S. Defending Comprehensive Tobacco Control Policy Implementation in Nepal From Tobacco Industry Interference (2011–2018). *Nicotine & Tobacco Research.* 2020;22(12):2203-2212. doi:10.1093/ntr/ntaa067

120. Wanyonyi MT, M. Kirigwajo, T. Rusoke. *Africa Tobacco Industry Monitoring (ATIM) Country Report: Uganda.* Sefako: Africa Tobacco Industry Monitoring (ATIM) 2020.

121. Hernández-Aguado I, Chilet-Rosell E. Pathways of undue influence in health policy-making: a main actor's perspective. *Journal of Epidemiology and Community Health.* 2018;72(2):154. doi:10.1136/jech-2017-209677

122. Illicit Tobacco Trade. Tobacco Tactics; 2021. Accessed 12 September 2021.

123. Freudenberg N. ToxicDocs: a new resource for assessing the impact of corporate practices on health. *Journal of Public Health Policy.* 2018;39(1):30-33. doi:10.1057/s41271-017-0101-0

124. Rosenstock L, Lee LJ. Attacks on Science: The Risks to Evidence-Based Policy. *American Journal of Public Health.* 2002;92(1):14-18. doi:10.2105/AJPH.92.1.14

125. University of Bath. Tobacco Tactics. 2020; https://tobaccotactics.org/topics/illicit/. Accessed 05 Feb, 2020.

126. Afshin A, Sur PJ, Fay KA, et al. Health effects of dietary risks in 195 countries, 1990–2017: a systematic analysis for the Global Burden of Disease Study 2017. *The Lancet.* 2019;393(10184):1958-1972. doi:https://doi.org/10.1016/S0140-6736(19)30041-8

127. Afshin A, Sur PJ, Fay KA, et al. Health effects of dietary risks in 195 countries, 1990–2017: a systematic analysis for the Global Burden of Disease Study 2017. *The Lancet.* 2019;393(10184):1958-1972. doi:10.1016/S0140-6736(19)30041-8

128. World Health Organisation. *Fiscal policies for diet and prevention of noncommunicable diseases: technical meeting repor.* Geneva: World Health Organisation,;2015.

129. Anaf J, Baum F, Fisher M, London L. The health impacts of extractive industry transnational corporations: a study of Rio Tinto in Australia and Southern Africa. *Global Health.* 2019;15(1):13. doi:10.1186/s12992-019-0453-2

130. Mialon M, Swinburn B, Wate J, Tukana I, Sacks G. Analysis of the corporate political activity of major food industry actors in Fiji. *Global Health.* 2016;12(1):18. doi:10.1186/s12992-016-0158-8

131. de Lacy-Vawdon C, Livingstone C. Defining the commercial determinants of health: a systematic review. *BMC Public Health.* 2020;20(1):1022. doi:10.1186/s12889-020-09126-1

132. Baker P, Russ K, Kang M, et al. Globalization, first-foods systems transformations and corporate power: a synthesis of literature and data on the market and political practices of the transnational baby food industry. *Global Health.* 2021;17(1):58. doi:10.1186/s12992-021-00708-1

133. Paichadze N, Werbick M, Ndebele P, Bari I, Hyder AA. Commercial determinants of health: a proposed research agenda. *Int J Public Health.* 2020;65(7):1147-1149. doi:10.1007/s00038-020-01445-9

134. Barata Cavalcanti O, Costa SA, Ferris E, et al. Benchmarking food and beverage company investment in healthful eating and active living initiatives. *Corporate Social Responsibility and Environmental Management.* 2020;27(2):1051-1068. doi:10.1002/csr.1865

135. Swinburn B, Vandevijvere S, Kraak V, et al. Monitoring and benchmarking government policies and actions to improve the healthiness of food environments: a proposed Government Healthy Food Environment Policy Index. *Obes Rev.* 2013;14 Suppl 1:24-37. doi:10.1111/obr.12073

136. Lee K, Freudenberg N. Public Health Roles in Addressing Commercial Determinants of Health. *Annual Review of Public Health.* 2022;43(1):375-395. doi:10.1146/annurev-publhealth-052220-020447

137. Lee K, Freudenberg N, Zenone M, et al. Measuring the Commercial Determinants of Health and Disease: A Proposed Framework. *International Journal of Health Services.* 2021;52(1):115-128. doi:10.1177/00207314211044992

138. Costa H, Gilmore AB, Peeters S, McKee M, Stuckler D. Quantifying the influence of the tobacco industry on EU governance: automated content analysis of the EU Tobacco Products Directive. *Tob Control.* 2014;23(6):473-478. doi:10.1136/tobaccocontrol-2014-051822

139. Gohil S, Vuik S, Darzi A. Sentiment Analysis of Health Care Tweets: Review of the Methods Used. *JMIR Public Health Surveill.* 2018;4(2):e43. doi:10.2196/publichealth.5789

140. Olstad DL, Lee J. Leveraging artificial intelligence to monitor unhealthy food and brand marketing to children on digital media. *Lancet Child Adolesc Health.* 2020;4(6):418-420. doi:10.1016/S2352-4642(20)30101-2

141. Sargent JD, Babor TF. The Relationship Between Exposure to Alcohol Marketing and Underage Drinking Is Causal. *Journal of studies on alcohol and drugs. Supplement.* 2020;Sup 19(Suppl 19):113-124. doi:10.15288/jsads.2020.s19.113

142. Madureira Lima J, Galea S. The Corporate Permeation Index - A tool to study the macrosocial determinants of Non-Communicable Disease. *SSM Popul Health.* 2019;7:100361. doi:10.1016/j.ssmph.2019.100361

143. Novotny TE, Bialous SA, Burt L, et al. The environmental and health impacts of tobacco agriculture, cigarette manufacture and consumption. *Bulletin of the World Health Organization.* 2015;93(12):877-880. doi:10.2471/BLT.15.152744

144. Topp SM, Schaaf M, Sriram V, et al. Power analysis in health policy and systems research: a guide to research conceptualisation. *BMJ Glob Health.* 2021;6(11). doi:10.1136/bmjgh-2021-007268
